# The effect of dexmedetomidine and clonidine on the inflammatory response in critical illness: a systematic review of animal and human studies

**DOI:** 10.1186/s13054-019-2690-4

**Published:** 2019-12-11

**Authors:** Charles A. Flanders, Alistair S. Rocke, Stuart A. Edwardson, J. Kenneth Baillie, Timothy S. Walsh

**Affiliations:** 1 Critical Care Department, Royal Infirmary of Edinburgh, Edinburgh, UK; 2 Department of Anaesthesia, Critical Care and Pain Medicine, University of Edinburgh, Edinburgh, UK; 3 The Royal Infirmary of Edinburgh, NHS Lothian, Edinburgh, UK

**Keywords:** Dexmedetomidine, Clonidine, Inflammation, Sepsis, Immune system, Critical care

## Abstract

**Background:**

The α2 agonists, dexmedetomidine and clonidine, are used as sedative drugs during critical illness. These drugs may have anti-inflammatory effects, which might be relevant to critical illness, but a systematic review of published literature has not been published. We reviewed animal and human studies relevant to critical illness to summarise the evidence for an anti-inflammatory effect from α2 agonists.

**Methods:**

We searched PubMed, the Cochrane library, and Medline. Animal and human studies published in English were included. Broad search terms were used: dexmedetomidine or clonidine, sepsis, and inflammation. Reference lists were screened for additional publications. Titles and abstracts were screened independently by two reviewers and full-text articles obtained for potentially eligible studies. Data extraction used a bespoke template given study diversity, and quality assessment was qualitative.

**Results:**

Study diversity meant meta-analysis was not feasible so descriptive synthesis was undertaken. We identified 30 animal studies (caecal ligation/puncture (9), lipopolysaccharide (14), acute lung injury (5), and ischaemia-reperfusion syndrome (5)), and 9 human studies. Most animal (26 dexmedetomidine, 4 clonidine) and all human studies used dexmedetomidine. In animal studies, α2 agonists reduced serum and/or tissue TNFα (20 studies), IL-6 (17 studies), IL-1β (7 studies), NFκB (6 studies), TLR4 (6 studies), and a range of other mediators. Timing and doses varied widely, but in many cases were not directly relevant to human sedation use. In human studies, dexmedetomidine reduced CRP (4 studies), TNFα (5 studies), IL-6 (6 studies), IL-1β (3 studies), and altered several other mediators. Most studies were small and low quality. No studies related effects to clinical outcomes.

**Conclusion:**

Evidence supports potential anti-inflammatory effects from α2 agonists, but the relevance to clinically important outcomes is uncertain. Further work should explore whether dose relationships with inflammation and clinical outcomes are present which might be separate from sedation-mediated effects.

## Background

The α2 agonists dexmedetomidine and clonidine are widely used as sedative drugs during critical illness, acting by dose-dependent decrease in activity of noradrenergic neurons in the brain stem via post-synaptic receptor-mediated inhibition [1]. This increases gamma-aminobutyric acid (GABA) neurone activity, which mediates central sedative effects. Dexmedetomidine, a highly selective α2 agonist (α2:α1 receptor selectivity ratio 1620:1), is licenced for intensive care unit (ICU) sedation and systematic reviews suggest it decreases duration of mechanical ventilation and ICU stay compared with propofol and/or benzodiazepines [2–4]. Effects on delirium are less certain [2, 4], but recent trials suggest dexmedetomidine can decrease delirium in selected populations, even when used in low doses [5]. Clonidine has substantially lower α2:α1 selectivity (220:1), is unlicensed for ICU sedation, but is also widely used in some countries. Despite this popularity, evidence for clinical effectiveness is very limited and of poor quality [6].

α2 agonists decrease central sympathetic nerve activity, which might affect inflammation and immune function either directly via cell surface receptors or indirectly by altering sympathetic/parasympathetic balance [7–9]. Although α2 agonists cause bradycardia, they may paradoxically increase cardiovascular stability in shock [10–12]. A post hoc trial analysis [13] and prospective clinical trial [14] suggest trends towards improved survival in sepsis when dexmedetomidine is used for sedation but remains unproven and was not observed in the recent large SPICE III randomised trial [15].

If α2 agonists have effects via mechanisms other than sedation, these could be important for clinical practice. Any anti-inflammatory effects could plausibly explain putative effects on delirium, shock, and other organ dysfunction and might explain why low doses of dexmedetomidine decrease delirium in perioperative patients [16]. Current evidence for anti-inflammatory effects from α2 agonists in critical illness has not previously been summarised. We therefore undertook a systematic review of relevant animal and human studies of critical illness to summarise current evidence.

## Methods

### Inclusion criteria

Eligible studies were animal or human studies undertaken in the context of sepsis, inflammation, or both. We limited studies to those that included dexmedetomidine or clonidine. All included studies required a biological measure of the inflammatory response as an outcome.

### Search

We searched PubMed, the Cochrane library, and Medline in accordance with the PRISMA guidelines; the PRISMA checklist is available as an Additional file 1. The final search was done on May 14, 2019. Search terms used were broad: dexmedetomidine, sepsis and inflammation; and clonidine, sepsis and inflammation. We had no date limits but included only studies published in English. Reference lists were screened for additional publications. After removing duplicates, titles and abstracts were screened independently by two reviewers (CAF and ASR) and full-text articles obtained wherever possible for all potentially eligible studies. Disagreements were resolved between the two reviewers, with reference to the senior author (TSW). Full-text articles were retrieved from the National Health Service, Athens, Knowledge Network, University of Edinburgh, and the British Library. Where a full-text article was unavailable and there was insufficient information in the abstract to determine eligibility, it was excluded.

For eligible studies, data were extracted into a table that summarised: type of study (animal, human), population, interventions used and comparators, outcomes, key summary findings, and any relevant additional comments or findings (see Additional files 2, 3, 4 and 5).

We grouped studies as follows:

#### Animal studies

Sepsis models (caecal ligation and puncture (CLP), and lipopolysaccharide (LPS)), acute lung injury models (ALI), and ischaemia/reperfusion injury (IRI) models.

#### Human studies

Any human study involving perioperative and/or critically ill patients fulfilling inclusion criteria.

As the type and range of studies varied widely and were mostly animal models, we did not undertake formal quality assessment using an existing tool. Instead, we documented potentially important limitations qualitatively for each study. Evidence synthesis was descriptive, because data were not suitable for meta-analysis.

## Results

**Fig. 1:**
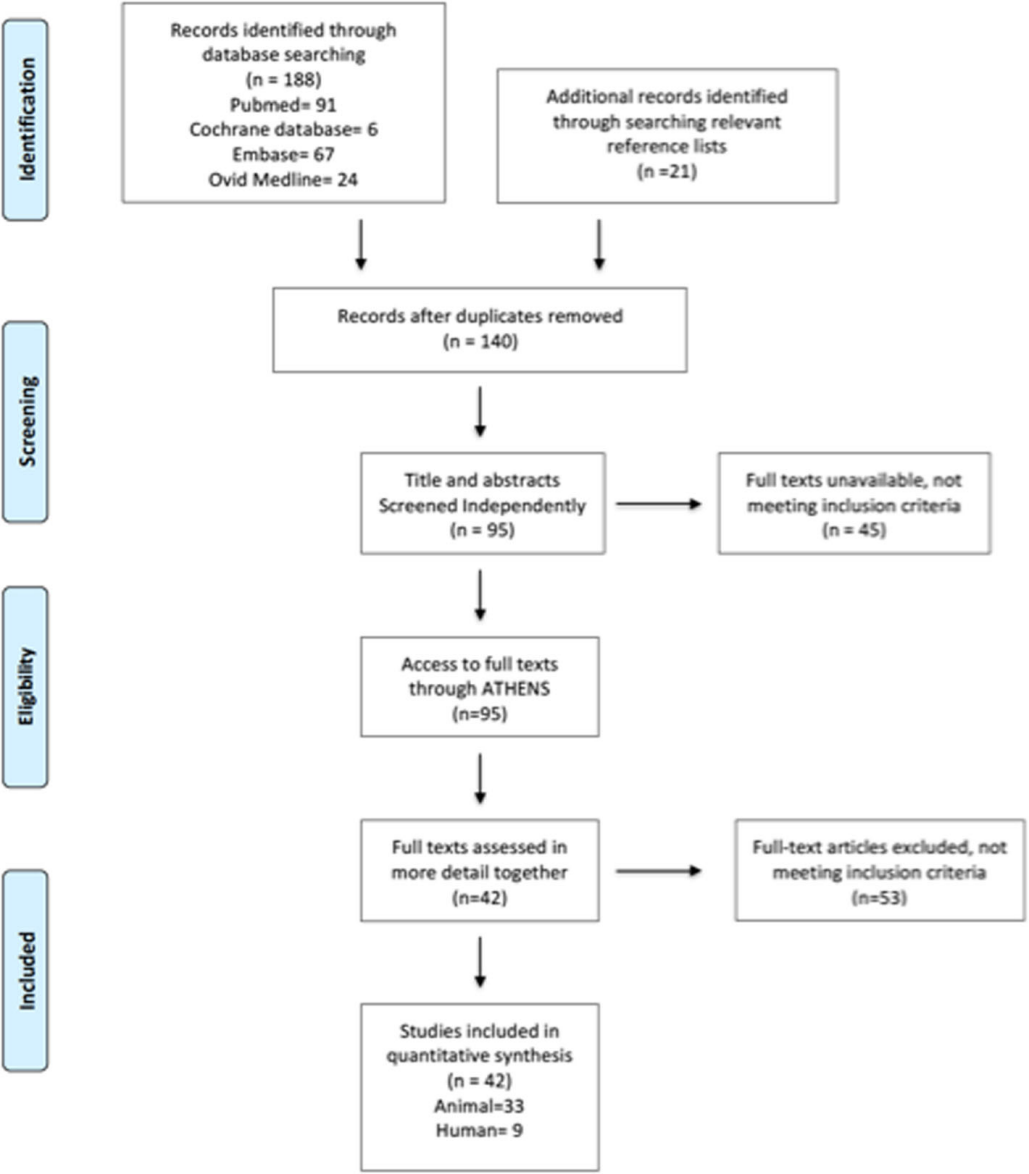
Systematic review PRISMA flowchart

The search identified 188 records; manual reference searching identified an additional 21 records. After removing duplicates 140 study abstracts were screened, and 95 selected for full text analysis. After full text review, 53 articles did not meet inclusion criteria and 42 articles were included in the review. The PRISMA flowchart is shown in Fig. 1. We identified 33 animal studies (9 CLP, 14 LPS, 5 ALI, 5 IRI) and 9 human studies.

### Animal studies

The key inflammatory molecules tested are described in Table 1 with a summary of all animal studies in Additional file 2 and a detailed description in Additional file 3.

**Table 1:** Inflammatory molecules tested in animal studies

Animal studies			
Inflammatory molecule	Alteration with α2 agonist	Location	Citation
Myeloperoxidase	Decreased	Lung tissue Bronchoalveolar lavage fluid Epigastric skin tissue flap	Chen et al. [17] Jiang et al. [18] Uysal et al. [19]
Nuclear factor Kappa B	Decreased	Lung tissue Bronchoalveolar lavage fluid Myocardial tissue	Chen et al. [17] Kong et al. [20] Hofer et al. [21] Wu et al. [22] Zhang et al. [23] Shen et al. [24]
Nitric Oxide/inducible nitric oxide synthase mRNA	Decreased	Lung tissue Serum Epigastric skin tissue flap	Chen et al. [17] Yang et al. [25] Yang et al. [26] Sugita et al. [27] Uysal et al. [19]
Tumour Necrosis Factor- α	Decreased	Lung tissue Bronchoalveolar lavage fluid Serum Myocardial tissue	Jiang et al. [18] Loftus et al. [28] Yang et al. [26] Chen et al. [3] Kang et al. [29] Kong et al. [20] Tan et al. [30] Taniguchi et al. [31] Taniguchi et al. [32] Xiang et al. [33] Chen et al. [17] Hofer et al. [21] Qiao et al. [34] Wu et al. [22] Feng et al. [35] Xu et al. [36] Zhang et al. [23] Shen et al. [24] Sugita et al. [27] Zhang et al. [4]
Interleukin-6	Decreased	Lung tissue Bronchoalveolar lavage fluid Serum Myocardial tissue	Jiang et al. [18] Yang et al. [25] Chen et al. [17] Kong et al. [20] Tan et al. [30] Feng et al. [35] Taniguchi et al. [31] Taniguchi et al. Xiang et al. [32] Chen et al. [3] Hofer et al. [21] Kang et al. [29] Qiao et al. [34] Zhang et al. [37] Wu et al. [22] Xu et al. [36] Zhang et al. [23] Shen et al. [24] Sugita et al. [27] Zhang et al. [4]
Toll-like Receptor 4	Decreased	Lung tissue Intestinal tissue Myocardial tissue	Jiang et al. [18] Chen et al. [17] Wu et al. [22] Zhang et al. [23] Shen et al. [24] Zhang et al. [4]
Myeloid Differentiation Primary Response 88 (MyD88)	Decreased	Lung tissue Myocardial tissue	Jiang et al. [18] Wu et al. [22] Zhang et al. [23] Zhang et al. [4]
Interleukin-10	Decreased	Serum	Szelenyi et al. [38]
Vascular endothelial growth factor	Increased	Lung tissue	Loftus et al. [28]
Vascular endothelial growth factor receptors 1 and 2	Increased	Lung tissue	Loftus et al. [28]
Interleukin-1b	Decreased	Lung tissue Serum Myocardial tissue	Yang et al. [25] Yang et al. [26] Kong et al. [20] Zhang et al. [37] Xiang et al. [33] Feng et al. [35] Chen et al. [17] Hofer et al. [21]
Α-7 nicotinic acetylcholine receptor	Increased	Myocardial tissue	Kong et al. [20]
Organic hyperoxides and superoxide radicals	Decreased	Lung tissue Hepatic tissue Ileal tissue	Chen et al. [17] Filos et al. [39]
High Mobility Group Box 1 (HMGB1)	Decreased	Lung tissue Renal tissue Myocardial tissue Serum	Loftus et al. [28] Tan et al. [30] Xu et al. [36] Zhang et al. [4]
Kidney Injury Molecule 1(KIM1)	Decreased	Renal tissue	Tan et al. [30]
B-Actin	Decreased	Serum	Yang et al. [25]
Cyclo-oxygenase 2 (COX-2)	Decreased	Serum	Yang et al. [25] Yang et al. [26]
Prostaglandin E2 (PGE2)	Decreased	Serum	Yang et al. [26]
Macrophage inflammatory protein 2 (MIP2)	Decreased	Serum	Yang et al. [26]
Lactate	Decreased	Serum	Chen et al. [17] Miranda et al. [40] Chen et al. [3]
Malondialdehyde (MDA)	Decreased	Renal tissue Epigastric skin tissue flap	Chen et al. [3] Koca et al. [41] Uysal et al. [19]

### Caecal ligation and puncture (CLP)

The nine CLP models were undertaken in rats [17, 21–23, 34, 41, 42] and mice [36, 37]. Seven studies used dexmedetomidine [17, 22, 23, 34, 36, 37, 41, 42] and one clonidine [21]. Study size ranged from 21 to 210 animals with a range of control group designs. Routes of administration were intraperitoneal [22, 36, 41, 42] and intravenous [17, 21, 23, 34, 37] as well as bolus [21–23, 36, 37, 41, 42] versus infusion [17, 34]. Study design included pre-only groups [34, 42], pre/post groups [21, 36], and post-only [17, 22, 23, 37, 41] designs. Three studies included α2 agonist blockade groups [17, 23, 42]. A range of dexmedetomidine doses was used but in most studies was high compared to human sedative doses. Duration of experiments varied widely (6 h to 7 days), but most were less than 24 h with widely varying sampling schedules.

Hofer et al. found both high dose intraperitoneal clonidine (5 μg/kg) and dexmedetomidine (40 μg/kg) decreased NFκB and pro-inflammatory cytokines (TNFα, IL-1β, IL-6) and improved survival [21], with effects most marked with pre-CLP administration. Qiao et al. found dexmedetomidine (5 μg/kg/h infusion) reduced Tumour necrosis factor alpha (TNFα), Interleukin-6 (IL-6), and caspase-3 but found similar effects from midazolam [34]. Wu et al. found higher doses of intraperitoneal dexmedetomidine (range 10–20 μg/kg) reduced inflammatory response within the lungs (bronchoalveolar lavage [BAL] TNFα, IL-6; lung tissue Toll-like receptor 4 (TLR4), Myeloid differentiation primary response 88 (MyD88), and NFκB) and improved survival [22]. Xu et al. found intravenous dexmedetomidine bolus (40 μg/kg) decreased TNFα and IL-6, with greatest effects after 24 h, and improved survival. Effects were greatest with pre-CLP dexmedetomidine administration [36]. Koca et al. found intraperitoneal dexmedetomidine (50 μg/kg post-CLP) decreased acute kidney injury (histology, creatinine, and Neutrophil gelatinase-associated lipocalin (NGAL)) and inflammatory markers (Cytokeratin 18 (CK18); M30) 6 h post-injury [41]. Zhang et al. found intravenous dexmedetomidine (5 or 10 μg/kg bolus post-CLP) attenuated all measured inflammatory markers (TNFα, IL-6, TLR4, MyD88, and NFκB) 6 h post-injury, but effects were not reduced by yohimbine [23]. Chen et al. found intravenous dexmedetomidine (5 μg/kg over 1 h) attenuated all inflammatory markers (TNFα, Interleukin-1β (IL-1β), IL-6, TLR4) and lactate production when delivered 30 min post-CLP, and improved survival; these effects were partially prevented by yohimbine [17]. Zhang et al. found intravenous dexmedetomidine at various doses (0.1, 0.3, 0.5 mg/kg) given 30 min post-CLP improved serum IL-6 and IL-1β [37]. Finally, Zhang et al. found dexmedetomidine (10 μg/kg) improved survival and histological lung injury scores (including tissue caveolin-1 expression), with effects partially reversed by the antagonist APZ [43].

### Lipopolysaccharide (LPS) administration

The 14 LPS models were undertaken in rats [30–33, 35, 44–47], mice [20, 29, 38, 48], and hamsters [40]. Thirteen studies used dexmedetomidine [20, 22, 29–33, 35, 40, 44–47] and one clonidine [38]. Study size ranged from 40 to 104 animals. Design varied widely and included pre-LPS only [20, 29, 30, 32, 33, 35, 38], post-LPS only [31, 40, 44–46, 48], and at initiation of LPS [47]. LPS was administered intravenously in all studies. Five studies [29, 30, 35, 38, 44] included comparison with α2 antagonist treatment. Doses (2.5 to 50 μg/kg) and duration of experiments (range 3 to 20 h) varied widely but most were short duration.

Taniguchi et al. found intravenous dexmedetomidine 5 μg/kg/h post-LPS decreased TNFα and IL-6, improved survival, and decreased lung neutrophil infiltration over 8 h [31]. A further study described dose-dependent reduction (dexmedetomidine 2.5 to 10 μg/kg/h) in TNFα and IL-6 over 5 h when started pre-LPS administration; effects were absent with lowest doses, and post-LPS administration had less marked effects [32]. Sezer et al. found intravenous dexmedetomidine 5 μg/kg/h decreased histological markers of inflammation 8 h post-LPS [45]. Shi et al. administered three boluses of dexmedetomidine (0.5, 1.5, and 4.5 μg/kg) and found lung TNFα, IL-6, IL-1β, TLR4, NFκB, and lung wet/dry ratios were all decreased with the two higher (but not the lowest) dexmedetomidine doses [46]. Kong et al. found dexmedetomidine administered as a 10 mg/kg bolus 1 h prior to LPS reduced myocardial apoptosis, NFκB, IL-6, and IL-1β and was attenuated by α-bungarotoxin (ABT), an antagonist at the α-7 nicotinic acetylcholine receptor [20]. Szelenyi et al. found intraperitoneal clonidine (5 mg/kg), administered 30 min prior to LPS, significantly reduced Interleukin-10 (IL-10) and was reversed when α2 antagonists (CH- 38083, WB04101, Prazosin) were administered [38]. Yeh et al. found intravenous dexmedetomidine (5 μg/kg/h) administered concurrently with LPS showed no harmful effect on cardiovascular parameters and reduced small bowel markers of endothelial dysfunction [47]. Wu et al. found dexmedetomidine 5 and 50 μg/kg/h infusion for 8 h from LPS administration attenuated T and B cell production, reduced macrophage phagocytosis at both doses, and increased NK cell activity at the high dexmedetomidine dose [48]. Xiang et al. administered dexmedetomidine 40 μg/kg as a bolus pre-LPS and found attenuation of TNFα, IL-6, and IL-1β and improved survival; effects were reversed by administration of the α2 antagonist ABT [33]. Chen et al. found intravenous dexmedetomidine 5 μg/kg/h attenuated a range of blood and liver inflammatory/injury markers, which was attenuated by yohimbine [44]. Miranda et al. used a skinfold chamber technique to study microcirculation in a hamster LPS model of dexmedetomidine 5 μg/kg/h 1 h post-LPS, finding no difference in vasodilatation, but a reduction in leukocyte rolling following dexmedetomidine [40]. Feng et al. administered dexmedetomidine at 25 μg/kg as a bolus pre-LPS and found attenuation of TNFα, IL-6, IL-8, and IL-1β. This effect was reversed when administered with ATZ [35]. Kang et al. found that dexmedetomidine administered at 40 μg/kg attenuated serum TNFα and IL-6 when given 1-h pre-LPS induced acute kidney injury. This effect was reversed when administered with the α2 antagonist ABT [29]. Finally, Tan et al. observed a decrease in TNFα, IL-6, Interleukin-8 (IL-8), and in renal High mobility group protein B1 (HMGB1) expression following pre-LPS dexmedetomidine 10 μg/kg administration, which was attenuated by concurrent yohimbine administration [30].

### Acute lung injury models

Five studies were undertaken in rats [18, 25, 26, 28] and dogs [49]. Four used dexmedetomidine [18, 25, 26, 49] and one clonidine [28]. Study size ranged from 30 to 84 animals. The ALI model was ventilator-induced lung injury (VILI) [25, 26, 49], ischaemia [18], and trauma [28]. Two studies [18, 25] included α2 agonist blockade groups. The dose varied widely 0.5 to 5 μg/kg/h dexmedetomidine and duration of experiments from 4 h to 7 days.

Yang et al. used a rat VILI model (10 versus 20 mL/kg) and compared effects from 0.5, 2.5, and 5 μg/kg dexmedetomidine. Lung histology studies and expression of TNFα, IL-1β, IL-6, macrophage inflammatory protein 2 (MIP-2), Nitric Oxide (NO), Prostaglandin E2 (PGE2), Nitric oxide synthase (iNOS), Cyclooxygenase 2 (COX2), and β-actin showed only the 5 μg/kg dose attenuated inflammation; the effect was attenuated by yohimbine [25]. In a further study, this group found intravenous dexmedetomidine at lower dose (0.5 μg/kg/h) reduced VILI in an LPS-plus-VILI model, based on histology and pro-inflammatory markers [26]. Chen et al. induced VILI in dogs (20 mL/kg), studied three intravenous dexmedetomidine doses from intubation (0.5, 1.0, and 2.0 μg/kg/h), and examined lung injury at 4 h. They found dose-dependent reduction in lung TNFα, iNOS, NFκB, and BAL Myeloperoxidase (MPO) and Polymorphonuclear neutrophils (PMN), with increasing dexmedetomidine dose [49]. Jiang et al. studied pre-injury dexmedetomidine (2.5 and 5 μg/kg/h) effects in an ischaemia (hilar occlusion) model of ALI in rats, finding dexmedetomidine reduced lung injury scores and inflammatory markers (MPO, TNFα, IL-6, TLR4, MyD88, Phospho-JNK, and Phospho-ERK); yohimbine partially reversed effects on some injury markers [18]. Finally, Loftus et al. used a complex trauma model including haemorrhage, lung contusion, and stress over 7 days to explore the effects of clonidine (10 μg/kg) on lung injury, finding high doses of clonidine increased vascular endothelial growth factor (VEGF) and its receptor expression in the lungs [28].

### Ischaemia-reperfusion injury (IRI) models

The five studies were all in rats, four dexmedetomidine [19, 24, 27, 43] and one clonidine [39]. Study size ranged from 30 to 80 animals. Two studies [24, 43] included α2 agonist blockade groups. Doses varied widely 0.5 to 5 μg/kg/h dexmedetomidine, as did duration of experiments (4 h to 7 days).

Filos et al. used a haemorrhage model and studied the effects of clonidine pre-treatment (150 μg/kg subcutaneously) on circulating endotoxin and tissue superoxide radicals, finding both were reduced [39]. Uysal et al. used a femoral artery/vein clamp model to study effects of dexmedetomidine 10 and 30 μg/kg on an epigastric island skin flap 12 h and 7 days post-IRI finding both doses reduced tissue NO, MDA, and MPO at both time points, and reduced flap necrosis area [19]. Shen et al. used a superior mesenteric artery occlusion model to study effects of dexmedetomidine 2.5 and 5 μg/kg/h infusion pre-IRI on lung injury, finding dose-dependent reductions in lung TLR4 and MyD88 (but not phosphoinositide 3-kinase (Pi3k) or Protein kinase B (AKT)) and improved lung histology and wet/dry weight ratio, with effects unaltered by yohimbine [24]. Sugita et al. studied effects of dexmedetomidine 10 and 20 μg/kg/h from the time of reperfusion on circulating TNFα, IL-6, IL-6, and NOS, finding attenuation of IL-6 and iNOS, but not TNFα, Endothelial derived nitric oxide (eNOS), or Neuronal nitric oxide (nNOS) 6 h post-IRI [27]. Finally, Zhang et al. studied the effects of dexmedetomidine infusion on myocardial injury in a coronary occlusion model, finding a reduction in infarct size and all measures of myocardial inflammation studied; this effect was attenuated by yohimbine [43].

### Human studies

We identified nine randomised controlled studies in humans. The populations were elective one-lung ventilation [50], laparoscopic cholecystectomy [51, 52], abdominal surgery [53, 54], spinal fusion surgery [55], cardiac surgery [56], and sepsis [14, 57]. All studies used dexmedetomidine; none used clonidine. Comparator treatments varied, comprising a control/placebo [50–52, 55, 56], propofol [14, 53, 54], and midazolam [57]. Size ranged from 20 to 201 patients; eight studies had a total size < 50 patients. Dexmedetomidine timing and dose varied: single bolus [50], in seven [51–57] as a bolus (range 0.5 to 2.5 μg/kg) followed by an infusion (range 0.5 to 2.5 μg/kg/h), and titration to a sedation target [14].

C Reactive Protein (CRP) was measured in four studies [14, 51, 55, 56] and was decreased in all studies compared to the comparator. TNFα was measured in five studies [50, 51, 53, 55, 57] and was reduced in all studies compared to the comparator. IL-6 was measured in six studies [51, 53–57]; dexmedetomidine decreased IL-6 in four studies [53, 55–57], but in two [51, 54] no difference was detected. IL-1β was measured in three studies [51, 53, 57] and was decreased in all studies. Other findings included a reduction in IL-10 [51], a transient attenuation of NFκB [56], an increase in the interferon/IL-4ratio [52], a decrease in CD42a/CD14 ratio and an increase in Human leukocyte antigen-DR isotype (HLA-DR)/CD14 ratio with dexmedetomidine [55]. The key inflammatory molecules tested are described in Table 2 with a summary of each study in Additional file 4 and a detailed description in Additional file 5.

**Table 2:** Inflammatory molecules tested in human studies

Human studies			
Inflammatory molecule	Alteration with α2 agonist	Location	Citation
Interferon Gamma	Increased	Serum	Kim et al. [52]
Tumour Necrosis Factor- α	Decreased	Serum	Gao et al. [50] Kang et al. [51] Memis et al. [57] Tasdogan et al. [53] Zhou et al. [55]
Superoxide Dismutase	Decreased	Serum	Gao et al. [50]
Malondialdehyde	Decreased	Serum	Gao et al. [50]
Heme-oxygenase 1	Decreased	Lung tissue	Gao et al. [50]
White Cell count	Decreased	Serum	Kang et al. [51] Zhou et al. [55]
Interleukin-1b	Decreased	Serum	Kang et al. [51] Memis et al. [57] Tasdogan et al. [53]
Interleukin-6	Decreased	Serum	Kang et al. [18] Memis et al. [57] Tasdogan et al. [53] Ueki et al. [56] Venn et al. [54] Zhou et al. [55]
Interleukin-10	Decreased	Serum	Kang et al. [51] Zhou et al. [55]
C Reactive Protein	Decreased	Serum	Kang et al. [51] Kawazoe et al. [14] Ueki et al. [56] Zhou et al. [55]
Procalcitonin	No statistically significant change	Serum	Kawazoe et al. [14]
Prealbumin	No statistically significant change	Serum	Kawazoe et al. [14]
Nuclear Factor Kappa B	Decrease	Serum	Ueki et al. [56]
High Mobility Group Box 1 (HMGB1)	Decrease	Serum	Ueki et al. [56]

## Discussion

We found both animal and human studies supported anti-inflammatory effects from the α2 agonists used in treatment of critically ill humans, namely dexmedetomidine and clonidine. However, only four animal and no human evaluated the effects of clonidine. There was wide variability in animal study design, but overall results were consistent with a reduction in markers of inflammation and organ injury. Anti-inflammatory effects were also observed in most human studies [14, 50, 51, 53, 55–57].

Animal studies were heterogeneous in design in relation to the stimulus (LPS, CLP, IRI, and VILI), route of administration and timing of α2 agonist, and dose regimen used. There was variation in the biological/pathological marker of inflammation used, but effects on pro-inflammatory cytokines were broadly consistent across studies. Studies using antagonists [4, 18, 20, 25, 29, 30, 33, 35, 38, 44] mostly suggested attenuation or elimination of effects, supporting a receptor-mediated mechanism, but this was not universal. The relevance of the animal studies to human disease is uncertain, especially because doses were mostly significantly higher than licenced for human use. No study measured plasma drug concentrations. Although the relevance to established critical illness of pre-insult administration is uncertain, this might be relevant to perioperative administration during major surgery. The co-administration of drugs such as ketamine in some studies [21, 26] might also interact with α2 agonist pathways, limiting relevance to human disease. Few studies measured concurrent cardiovascular responses [21, 27, 31, 32, 47], which are potentially important side effects in humans, and almost all studies were undertaken in small animals, which may not be relevant models to human inflammatory disease. The consistency of reported effects across studies supports potentially relevant modulation of inflammation and/or immune responses. However, we cannot exclude an influence from publication bias.

Of the nine human studies, only two were undertaken in critically ill patients with sepsis or other clear inflammatory conditions [14, 57]; the remainder included elective surgical populations [20, 50, 52–56]. Most studies performed serum cytokine assays until around 48 h post-operatively and observed reductions compared with control patients in the immediate post-operative period. The main study limitations were small sample size and the uncertain relevance of inflammatory mediator concentrations to clinical outcomes. All of the 9 human studies used dexmedetomidine. The largest study was undertaken in mechanically ventilated septic patients and had non-significantly different clinical primary outcomes (mortality and ventilation-free days). Dexmedetomidine doses were smaller than those widely used for clinical sedation (0.1–0.7 μg/kg/h), but despite this the observed reduction in CRP concentrations is consistent with an anti-inflammatory effect. This study did not measure pro- or anti-inflammatory pathways [14]. The size and quality of the human studies was generally low, populations were heterogeneous, and most populations did not have severe illness. Anti-inflammatory effects were observed for potentially relevant inflammatory mediators in all studies, but none linked modulation of inflammation to clinical outcomes.

This is the first systematic review of the anti-inflammatory effects of dexmedetomidine and clonidine that includes both human and animal studies. We included studies covering a wide range of inflammatory insults including animal sepsis, ARDS and ischemia models, human critical illness, and major surgical interventions. In animal studies, we also specifically described the dose of α2 agonist used and timing of administration to highlight relevance to human disease. The only other published systematic review described anti-inflammatory effects of perioperative dexmedetomidine as an adjunct to general anaesthesia and found significant reductions in perioperative IL-6, IL-8, TNFα concentrations, and post-operative IL-10. It also observed wide variation in the surgery studied and high statistical heterogeneity of effects between studies. However, the relevance to clinical outcomes was not included and only dexmedetomidine administered pre-operatively was reviewed [8]. Our review summarised the available animal data and also highlights the limited evidence for clonidine. Taken together, these data support a possible anti-inflammatory effect from α2 agonists but are inconclusive about the clinical importance of this finding.

There are several suggested mechanisms for an anti-inflammatory/immune regulatory effect from α2 agonists. These include modulation of macrophage/monocyte function through α2-receptor-mediated inhibition of apoptosis, central and peripheral α2-receptor agonist-mediated effects, direct or indirect central sympatholytic effects, stimulation of cholinergic anti-inflammatory pathways, reduction in anxiety and stress, and indirect effects via anti-nociceptive and/or sedative effects. The studies included in our review do not enable the exact mechanisms to be conclusively elucidated, but those that included an antagonist [4, 18, 20, 25, 29, 30, 33, 35, 38, 44] mostly showed attenuation of α2 receptor agonist-mediated anti-inflammatory/immune regulatory effects suggesting a receptor-mediated effect is an important mechanism. This, however, was not a universal finding.

Whether anti-inflammatory effects are on a causative pathway to improved clinical outcomes in human critical illness is unproven. In the recent SPICE trial, which compared early dexmedetomidine based sedation with usual care (mostly propofol and/or midazolam) no overall mortality benefit was found and there was also no mortality benefit in a large sepsis sub-group (in whom an anti-inflammatory benefit may be most biological plausible) [15]. However, there were beneficial effects on both ventilation duration and delirium, and in a sub-group analysis the authors observed differences in the effect on mortality between younger (greater mortality with dexmedetomidine) and older (lower mortality with dexmedetomidine) patients for which the significance is uncertain. The mechanism of differential age-related effects of these agents is unexplained, but non-sedation related actions potentially, including immune/inflammatory modulation, might contribute. Further human studies are needed to explore whether the anti-inflammatory properties of α2-agonists are clinically important and whether differences between dexmedetomidine and clonidine might influence their modulation of clinical outcomes.

Our review has several limitations. Firstly, we used broad search terms to try and capture a wide range of studies, but we may have missed potentially relevant papers. Second, we cannot exclude publication bias especially for animal studies. Third, quantitative meta-analysis was not undertaken, because we did not consider this plausible or relevant for the identified literature; our data synthesis was therefore descriptive.

## Conclusion

In conclusion, although the quality of animal and human studies is low, we have shown that α2 agonist drugs may potentially modify inflammatory and immune pathways in acute inflammatory conditions. Further work is required, especially in critically unwell humans, to explore mechanisms of action and whether these translate into improved patient outcomes.

## Supplementary information

**Additional file 1.** PRISMA Checklist.

**Additional file 2.** Summary of animal studies.

**Additional file 3.** Detailed description of animal studies.

**Additional file 4.** Summary of human studies.

**Additional file 5.** Detailed description of human studies.

## Data Availability

PubMed, the Cochrane library, and Medline databases

## Abbreviations

ABT: α-bungarotoxin
AKT: Protein kinase b
ALI: Acute lung injury
APZ: Atipamezole
ARDS: Acute respiratory distress syndrome
CK18: Cytokeratin 18
CLP: Caecal ligation and puncture
COX2: Cyclooxygenase 2
CRP: C Reactive protein
eNOS: Endothelial derived nitric oxide
GABA: Gamma-aminobutyric acid
HLA-DR: Human leukocyte antigen-DR isotype
HMGB1: High mobility group protein B1
ICU: Intensive Care unit
IL-1β: Interleukin-1β
IL-10: Interleukin-10
IL-6: Interleukin-6
IL-8: Interleukin-8
iNOS: Nitric Oxide Synthase
IRI: Ischaemia/reperfusion injury
LPS: Lipopolysaccharide
MIP-2: Macrophage inflammatory protein 2
MPO: Myeloperoxidase
MyD88: Myeloid differentiation primary response 88
NFκB: Nuclear factor kappa-light-chain-enhancer of activated B cells
NGAL: Neutrophil gelatinase-associated lipocalin
nNOS: Neuronal nitric oxide
NO: Nitric Oxide
PGE2: Prostaglandin E2
Pi3k: Phosphoinositide 3-kinase
PMN: Polymorphonuclear neutrophils
TLR4: Toll-like receptor 4
TNFα: Tumour necrosis factor alpha
VEGF: Vascular endothelial growth factor
VILI: Ventilator-associated lung injury
