# Supplementary material for: The effect of dexmedetomidine and clonidine on the inflammatory response in critical illness: a systematic review of animal and human studies
Source: Crit Care. 2019 Dec 11;23:402. doi: 10.1186/s13054-019-2690-4 (PMC6907244; doi:10.1186/s13054-019-2690-4)
Supplement: Supplementary file 2 — Additional file 2. Summary of animal studies. [file 13054_2019_2690_MOESM2_ESM.doc]

| **Author** | **Population** | **Intervention** | **Key Findings** | | **Main Conclusions** | |
| --- | --- | --- | --- | --- | --- | --- |
| **Lung Injury** |  |  |  | |  | |
| **Chen 2014** | Dogs | **Injury:** Ventilator induced lung injury (VILI)  **Study drug**: Dexmedetomidine (infusion, 0.5/1.0/2.0 micrograms/kg/hr)  **Study duration:** 4 hours | **1**.Dose dependent reduction in intra-alveolar exudates and inflammatory cell infiltration with increasing dexmedetomidine dose.  **2.**Lung tissue concentration of myeloperoxidase, polymorphonuclear neutrophils, nuclear factor-Kb (NFKb) messenger RNA (mRNA) and inducible nitric oxide synthase (iNOS) mRNA were all significantly reduced in the 1.0mcg/kg/hr and 2.0mcg/kg/hr dexmedetomidine groups. | | Pre-medication and infusion with dexmedetomidine at clinically relevant doses can reduce histological evidence of inflammation in the lung after VILI.  This effect seemed to be dose dependent. | |
| **Jiang 2014** | Rats | **Injury**: Ischaemia-reperfusion injury (IRI) of left lung  **Study drug:** Dexmedetomidine (infusion, 2.5/5.0 micrograms/kg/hr), yohimbine (alpha-2 antagonist).  **Study duration:** 4 hours | **1.**Dexmedetomidine pre-treatment showed reduction in inflammatory cell infiltration seen of lungs.  **2.**MPO, TNF-alpha, IL-6, TLR4 and MyD88 were all attenuated by dexmedetomidine.  **3.**P-JNK and P-ERK expression were both reduced by dexmedetomidine in a dose-dependent manner.  **4.**Yohimbine only partially reversed the anti-inflammatory effect of dexmedetomidine. | | Pre-treatment with dexmedetomidine may attenuate the pulmonary damage associated with IRI.  Only partial reversal was displayed with an alpha-2 antagonist. | |
| **Loftus 2017** | Rats | **Injury:** Traumatic insult (various methods)  **Study drug:** Clonidine (daily bolus, 75 micrograms/kg), propranolol (daily bolus, 10 mg/kg).  **Study duration:** 7 days | **1**.Daily clonidine administration was associated with increased VEGF and VEGF receptor expression. This was associated with reduced lung inflammation.  **2.**Propranolol had a similar, but less pronounced and less consistent anti-inflammatory effect.  **3.**TNF-alpha was reduced by both study medications, but more so by clonidine. | | In high doses, administered regularly after daily traumatic insult, clonidine is associated with increased VEGF and its receptor expression in the lungs. This effect is more pronounced with clonidine in comparison to propranolol. | |
| **Yang 2008** | Rats | **Injury:** VILI  **Study drug:** Dexmedetomidine (infusion, 0.5/2.5/5.0 micrograms/kg/hr), yohimbine.  **Study duration:** 5 hours | **1.**Dexmedetomidine infusion at 5.0micrograms/kg/hr attenuated lung injury score, inflammatory infiltration on lung histology, and expression of inflammatory molecules. This was not seen with any lower dose.  **2.** Yohimbine significantly reduced this anti-inflammatory effect | | No anti-inflammatory effect (histological or cytokine) was found at any dose lower than 5.0 micrograms/kg/hr.  Yohimbine caused significant reduction in this anti-inflammatory effect. | |
| **Yang 2011** | Rats | **Injury:** VILI, LPS-induced endotoxaemia, or both.  **Study drug:** Dexmedetomidine (infusion, 0.5 micrograms/kg/hr) co-administered with ketamine (infusion, 1mg/kg/hr)  **Study duration:** 4 hours | **1.**All groups receiving dexmedetomidine and ketamine combination therapy showed reduced histological evidence of lung damage and inflammation, and a reduction in measured circulating cytokines.  **2**.There was no difference seen between VILI, LPS, or VILI + LPS groups. | | The combination of dexmedetomidine and ketamine in clinically relevant doses may have an anti-inflammatory effect in models of VILI and endotoxaemia. | |
| **LPS** |  |  |  | |  | |
| **Chen 2015** | Rats | **Injury:** LPS-induced endotoxaemia  **Study drug:** Dexmedetomidine (5 micrograms/kg/hr infusion)  **Study duration:** 6 hours | **1.**Dexmedetomidine reduced histological evidence of liver inflammation and apoptosis. Serum AST, ALT, and lactate were also reduced.  **2.**Yohimbine co-administration completely reversed any beneficial effect of dexmedetomidine. | | Dexmedetomidine at 5 micrograms/kg/hr improves liver injury score after endotoxaemia is induced by LPS in rats.  This effect is completely reversed by administration of an alpha-2 antagonist. | |
| **Fen 2019** | Rats | **Injury:** LPS induced renal injury  **Study drug:** Dexmedetomidine (bolus 25micrograms/kg)  ATZ (250micrograms/kg)  IDA (1.5mg/kg)  **Study duration:** 4 hours | **1.** Dexmedetomidine reduced LPS induced renal dysfunction and histology.  **2.** Dexmedetomidine attenuated serum inflammatory cytokines  **3.** The effects of Dexmedetomidine were attenuated by the alpha-2 antagonists ATZ and IDA. | | Dexmedetomidine given as a bolus, prior to LPS attenuated renal dysfunction and inflammatory cytokines. This effect is reversed by administration of an alpha-2 antagonist. | |
| **Kang 2018** | Mice | **Injury:** LPS induced renal injury  **Study drug:** Dexmedetomidine (40micrograms/kg)  ABT (1 microgram/kg)  *ABT is an antagonist at the alpha-7 nicotinic acetylcholine receptor present in the central nervous system. | **1.** Dexmedetomidine attenuated LPS induced renal pathology and serum inflammatory cytokines.  **2.** This effect was revered when administered with ABT, an alpha-2 antagonist. | | Dexmedetomidine when administered as an intraperitoneal bolus pre-LPS attenuated renal pathology and serum inflammatory cytokines.  This effect was attenuated by ABT. | |
| **Kong 2017** | Mice | **Injury:** Septic cardiomyopathy induced by LPS.  **Study drug:** Dexmedetomidine (bolus, 10mg/kg), ABT (bolus, 1 microgram/kg).  **Study duration:** 16 hours. | **1**.Dexmedetomidine markedly reduced histological evidence of myocardial tissue damage. It was also associated with reduced tissue cytokine concentration, NFkB activity, and proteins associated with cellular apoptosis.  **2.**Alpha-7 nicotinic acetycholine receptor was decreased as a result of endotoxaemia, but increased when dexmedetomidine was administered. Administration of its antagonist (ABT) abolished the protective effects of dexmedetomidine. Myocardial apoptosis and all cytokines returned to pre-dexmedetomidine levels. | | Dexmedetomidine at 10mg/kg was associated with reduced myocardial apoptosis and inflammation.  Its protective effects on the myocardium may be through its action in the cholinergic pathway via alpha 7 nicotinic acetylcholine receptor. | |
| **Miranda 2015** | Hamsters | **Injury:**LPS-induced endotoxaemia  **Study drug:** Dexmedetomidine (infusion, 5 micrograms/kg/hr)  **Study duration:** 4 hours | **1.**Dexmedetomidine was associated with reduced microvascular dysfunction. There was evidence of attenuation of the reduced functional capillary density and reduced erythrocyte velocity seen in endotoxaemia.  **2.**Dexmedetomidine was associated with a reduction in heart rate, but not MAP.  **3.**Dexmedetomidine was associated with a reduction in lactate. | | Dexmedetomidine treatment at 5 micrograms/kg/hr may improve microcirculatory function in a hamster model of endotoxaemia. | |
| **Sezer 2010** | Rats | **Injury:** LPS-induced endotoxaemia  **Study drug:** Dexmedetomidine (infusion, 5 micrograms/kg/hr)  **Study duration:** 8 hours | **1.**Dexmedetomidine reduced histological appearance of hepatic injury. | | An infusion of dexmedetomidine at 5 micrograms/kg/hr is associated with reduced histological evidence of hepatic injury in a rat model of endotoxaemia. | |
| **Shi 2012** | Rats | **Injury:** LPS-induced Acute Lung Injury (ALI)  **Study drug:** Dexmedetomidine (bolus, 0.5 micrograms/kg, 1.5 micrograms/kg, 4.5 micrograms/kg)  **Study duration:** 6 hours | **1.**The 1.5 and 4.5 micrograms/kg groups showed reduced wet/dry ratio of the lungs, as well as reduced inflammatory cell infiltration, and alveolar wall thickening. These effects were not seen in the lowest dose treatment group.  **2.** The 1.5 and 4.5 micrograms/kg groups showed a reduction in NFkB expression. This was the same for TLR4 mRNA expression and cytokine concentration. | | Dexmedetomidine at 1.5 micrograms/kg and 4.5 micrograms/kg bolus can reduce evidence of ALI induced by LPS in a dose-dependent manner. | |
| **Szelenyi 2000** | Mice | **Injury:** LPS-induced endotoxaemia  **Study drug:**  **Alpha-2 agonist groups**  UK 14304 (5mg/kg)  Xylazine (5mg/kg)  Clonidine (5mg/kg)  **Alpha-2 antagonist groups**  CH- 38083 (10mg/kg)  WB-4101 (10mg/kg)  Prazosin (5mg/kg)  **Study duration:** 90 minutes | **1.**Alpha-2 agonists caused a significant reduction in serum IL-10 in comparison to LPS only groups.  **2.**Alpha-2 antagonists caused a significant increase in serum IL-10 in comparison to LPS only groups. | | Alpha-2 agonists may be associated with reduced IL-10 levels at 90 minutes after LPS administration in mice. | |
| **Tan 2015** | Rats | **Injury:** LPS-induced endotoxaemia  **Study drug:** Dexmedetomidine (bolus, 10 micrograms/kg), yohimbine (1mg/kg).  **Study duration:** 4 hours | **1.**Dexmedetomidine was associated with reduced occurrence of acute kidney injury during LPS-induced endotoxaemia.    **2.** Dexmedetomidine was associated with reduced histological evidence of renal damage. This included reduced inflammatory cell infiltration and epithelial cell degeneration.  **3.**All measured serum inflammatory markers were reduced when the dexmedetomidine groups.  **4.**Yohimbine co-administration reduced any of dexmedetomidine’s effects on acute kidney injury, histopathology, and circulating cytokine levels. | | Pre-treatment with a bolus of 10 micrograms/kg of dexmedetomidine is associated with reduced renal injury in a rat model of LPS-induced endotoxaemia. | |
| **Taniguchi 2004** | Rats | **Injury:** LPS-induced endotoxaemia  **Study drug:** Dexmedetomidine (infusion, 5 micrograms/kg/hr)  **Study duration:** 8 hours | **1.**Dexmedetomidine infusion was associated with reduced 8h mortality from 94% to 44%.  **2.** Dexmedetomidine infusion was associated with almost complete absence of hypotension seen in the LPS only groups.  **3.** Dexmedetomidine infusion was associated with reduced inflammatory cell infiltration and gross signs of injury in the lungs. | | Dexmedetomidine infusion at 5 micrograms/kg/hr was associated with reduced global markers of inflammation including plasma cytokines and lung inflammation, in a rat model of LPS-induced endotoxaemia.  Dexmedetomidine was also associated with reduced mortality and incidence of hypotension. | |
| **Taniguchi 2008** | Rats | **Injury:** LPS-induced endotoxaemia  **Study drug:** Dexmedetomidine (infusion, 2.5/5/10 micrograms/kg/hr).  **Study duration:** 8 hours  Some further test groups were given dexmedetomidine 10 micrograms/kg/hr at varying times after LPS injection to assess for any time-related differences seen. | **1**.All groups receiving dexmedetomidine showed improved blood pressure in comparison to the hypotension seen with LPS only groups (with exception of delayed dexmedetomidine administration 2 hours after LPS).    **2.**All groups that received dexmedetomidine had significantly lower mortality rates than those which did not. This benefit was the most modest in the group receiving delayed dexmedetomidine 2h after LPS.  **3.** Plasma TNF-alpha and IL-6 were reduced in all groups that received dexmedetomidine. This was dose-dependent. Delayed dexmedetomidine 2h after LPS showed only a modest decrease in both inflammatory markers. | | In a model of LPS-induced endotoxaemia in rats, dexmedetomidine pre-treatment shows a safe cardiovascular profile, with a dose-dependent reduction in plasma inflammatory markers and mortality.  Delayed administration of dexmedetomidine (2h) produces only modest benefit in comparison to early administration (1h). | |
| **Wu 2014** | Mice | **Injury:** LPS-induced endotoxaemia  **Study drug:** Dexmedetomidine (infusion, 5 micrograms/kg/hr, 50 micrograms/kg/hr).  **Study duration:** 8 hours | **1.**Low dose dexmedetomidine reduced numbers of peripheral T cells and B cells in whole blood, blunting the increase caused by LPS. It had no effect on peripheral numbers of NK cells, macrophages, and monocytes.  High dose dexmedetomidine had no further effect on peripheral T cell and B cell numbers, but did decrease numbers of macrophages seen.  **2.**Dexmedetomidine reduced systemic macrophage phagocytosis at low dose and high dose to the same degree.  **3.**Dexmedetomidine increased systemic NK cell activity, even more so at the higher dose.  **4.**‘Infection site’ samples showed preserved macrophage activity at low dose, and enhanced it at high dose. | | In a mouse model of LPS-induced endotoxaemia, 5 mcg/kg/hr infusion of dexmedetomidine decreases peripheral B cell and T cell augmentation, while preserving ‘infection site’ macrophage and systemic NK cell activity.  High dose dexmedetomidine enhanced NK cell and ‘infection site’ macrophage activity. | |
| **Xiang 2014** | Rats | **Injury:** LPS-induced endotoxaemia  **Study drug:** Dexmedetomidine (bolus, 40 micrograms/kg), ABT (bolus, 1 microgram/kg).  Some groups also underwent vagotomy via cervical vagus nerve  **Study duration:** 3h for cytokine analysis, 120h for survival analysis. | **1.**Dexmedetomidine was associated with significantly increased 120 hour survival (0.65 vs 0.25 in the control group)  **2.** Dexmedetomidine was associated with a significant reduction in all measured serum cytokines at 3 hours. This effect was abrogated by ABT co-administration.  **3.**Subjects receiving dexmedetomidine and vagotomy showed similar results to LPS only groups.  **4.** Dexmedetomidine was associated with significantly increased vagal nerve discharge frequency (346 Hz vs 179Hz in saline group), but had no effect on discharge amplitude. | | Dexmedetomidine pre-treatment is associated with increased survival and reduced plasma inflammatory markers in a rat model of LPS-induced endotoxaemia.  This anti-inflammatory effect is abrogated with both vagotomy and alpha-bungarotoxin. This suggests a role for the cholinergic pathway in the anti-inflammatory mechanism of dexmedetomidine. | |
| **Yeh 2016** | Rats | **Injury:** LPS-induced endotoxaemia  **Study drug:** Dexmedetomidine (infusion, 5 micrograms/kg/hr)  **Study duration:** 4 hours. | **1.**Dexmedetomidine was associated with no difference in measured blood pressure or heart rate.  **2.**Dexmedeotmidine was associated with improved small bowel microcirulatory flow, reduced endothelial dysfunction, attenuated intestinal epithelial cell death, and reduced intestinal bacterial translocation. | | Dexmedetomidine was associated with improvements in small bowel microcirculation and reduced markers of endothelial dysfunction in a rat model of LPS-induced endotoxaemia. | |
| **CLP** |  |  |  | |  | |
| **Chen 2015** | Rats | **Injury:** Caecal ligation and puncture (CLP).  **Study drug:** Dexmedetomidine (bolus, 5 micrograms/kg), yohimbine (1mg/kg).  **Study duration:** 24h for sample analysis, 7 days for survival analysis | **1.** Dexmedetomidine reduced 7-day mortality from 80% (CLP only) to 30% (CLP and dexmedetomidine). Co-administration of yohimbine increased mortality almost back to original rates (70%).  **2.**Dexmedetomidine was associated with amelioration of the intestinal damage caused by CLP.  **3.** Dexmedetomidine was associated with reduced concentrations of all serum inflammatory markers measured.  **4.**Dexmedetomidine was associated with decreased TLR-4 expression.  **5.** Yohimbine co-administration attenuated all anti-inflammatory effects seen with dexmedetomidine. | | Dexmedetomidine is associated with reduced serum inflammatory markers, intestinal TLR-4 expression, and histological evidence of intestinal damage in a rat model of CLP. | |
| **Hofer 2009** | Rats | **Injury:** CLP  **Study drug:** Clonidine (bolus, 5 micrograms/kg), dexmedetomidine (40 micrograms/kg)  Both drugs were given at regular intervals before and after CLP.  **Study duration:** 24 hours for samples, 5 days for survival analysis. | **1.**Both clonidine and dexmedetomidine groups showed a significant improvement in survival at 5 days. Post-CLP treatment only with clonidine showed a trend towards survival, but did not reach statistical significance.  2.Blood pressure was improved in the groups given clonidine pre-treatment.  2. All serum cytokine levels measured were reduced with clonidine pre-treatment. The same was seen for NFkB activity. | | Regular bolus of clonidine both before and after CLP showed improved 5 day mortality, improved blood pressure profile, and reduced serum inflammatory markers in a rat model.  Clonidine given only after CLP showed only modest improvements in these outcomes. | |
| **Koca 2013** | Rats | **Injury:** CLP  **Study drug:** Dexmedetomidine (bolus, 50 micrograms/kg)  **Study duration:** 6 hours | **1**.Dexmedetomidine treatment was associated with attenuation of all histological markers of both kidney and lung injury.  **2.**Dexmedetomidine was also associated with reduction in biochemical markers of kidney injury. | | A bolus of 50 micrograms/kg of dexmedetomidine immediately after CLP may reduce histological evidence of renal and lung injury alongside biochemical markers of kidney injury. | |
| **Qiao 2009** | Rats | **Injury:** CLP  **Study drug:** Midazolam (infusion, 0.6mg/kg/hr), dexmedetomidine (infusion, 5 micrograms/kg/hr).  **Study duration:** 32 hours | **1.**Mortality was reduced to a similar degree in both the midazolam and dexmedetomidine groups.  **2**.Both sedatives reduced serum levels of TNF-alpha, but only dexmedetomidine reduced levels of IL-6 (although not statistically significant).  **3.** Splenic caspase 3 (a marker of apoptosis) was reduced most markedly in the dexmedetomidine group. | | At a dose of 0.6mg/kg/hr midazolam and 5 micrograms/kg/hr dexmedetomidine, anti-inflammatory effects can be seen in a CLP rat model. Dexmedetomidine may have pronounced action by reducing IL-6 and apoptosis of splenic cells. | |
| **Wu 2013** | Rats | **Injury:** CLP  **Study drug:** Dexmedetomidine (bolus, 5/10/20 micrograms/kg).  **Study duration:** 24 hours  Dexmedetomidine bolus was given intraperitoneally at 0h, 2h, 4h, and 6h post CLP. | **1.**A dose dependent improvement in 24h mortality was seen with dexmedetomidine treatment.  **2**.Only medium and high dose dexmedetomidine had an anti-inflammatory effect on both serum and BAL cytokine measurements, as well as NFkB activity.  **3.**Only medium and high dose dexmedetomidine showed histological improvement in lung injury score. | | Dexmedetomidine bolus at 10 and 20 mirograms/kg showed a dose dependent anti-inflammatory effect on both systemic and lung markers of inflammation in a rat model of CLP. This also translated into a decrease in 24 hour mortality. | |
| **Xu 2013** | Mice | **Injury:** CLP  **Study drug:** Dexmedetomidine (bolus, 40 micrograms/kg)  One group was given this 1 hr before CLP, and another given the drug 1 hr after CLP.  **Study duration:** 48h for samples, 7 days for survival analysis. | 1.Both dexmedetomidine groups showed reduced mortality at 7 days. This effect was most pronounced in the pre-treatment group.  **2.** Dexmedetomidine was associated with attenuated cytokine levels at 24 hours post CLP. This was only slightly more marked in the pre-treatment group.  **3.** Serum HMGB1 and HMGB1 mRNA (lung) both followed the same pattern as serum cytokine expression. | | A bolus of 40 micrograms/kg of dexmedetomidine was associated with decreased 7 day mortality and 24 hour cytokine levels in a mouse model of CLP. This effect seemed to be greatest when the drug was given 1 hr before injury. | |
| **Zhang 2019** | Mice | **Injury:** CLP  **Study drug:** Dexmedetomidine (0.1/0.3/0.5mg/kg)  **Study duration:** | 1. Dexmedetomidine reduced lung injury score and attenuated serum inflammatory cytokines when given 30 minutes post- CLP.  2. Dexmedetomidine attenuates mitochondrial damage. | | Intravenous post-CLP dexmedetomidine improves the histological lung injury score and attenuates serum inflammatory cytokines. | |
| **Zhang 2015** | Rats | **Injury:** CLP  **Study drug:** Dexmedetomidine (bolus, 5/10 micrograms/kg/hr), yohimbine (1mg/kg)  **Study duration:** 6 hours | **1.**Dexmedetomidine was associated with reduced cytokine levels in serum and BAL at both doses. This was also the case for NFkB activity.  **2.**This anti-inflammatory effect was sustained even when co-administered with yohimbine. | | Intravenous bolus of 5 micrograms/kg and 10 micrograms/kg of dexmedetomidine after CLP showed a non-dose dependent reduction in serum and BAL cytokine levels.  This outcome was not affected by co-administration of yohimbine. | |
| **Zhang 2017** | Rats | **Injury:** CLP  **Study drug:** Dexmedetomidine (bolus, 10 micrograms/kg), atipamezole (1mg/kg)  **Study duration:** 24 hours | **1.**Dexmedetomidine was associated with increased 24 hour survival.  **2.**Dexmedetomidine was associated with improved lung injury score and histological markers of lung damage/inflammation.  **3**.Atipamezole (alpha-2 antagonist) abrogated this anti-inflammatory effect significantly. | | A bolus of 10 micrograms/kg of dexmedetomidine was associated with increased 24 hour survival and reduced lung injury score in a rat model of CLP.  This effect may be due to action via the alpha-2 receptor. | |
| **IRI** |  |  |  | |  | |
| **Filos 2012** | Rats | **Injury:** IRI via haemorrhage from femoral artery.  **Study drug:** Clonidine (subcutaneous bolus, 150 micrograms/kg). Given 5 times a day in the 2 days leading up to the experiment.  **Study duration:** 3 days | **1.**Clonidine pre-treatment was associated with reduced organic hydroperoxides and superoxide radicals found in all of the tissues sampled.  **2.** Clonidine pre-treatment was associated with reduced circulating endotoxin. | | Regular subcutaneous pre-treatment with 150 micrograms/kg of clonidine may reduce production of organic hydroperoxides and superoxide radicals along with circulating endotoxin in a rat model of haemorrhagic shock. | |
| **Shen 2013** | Rats | **Injury:** IRI was performed by clamping of the superior mesenteric artery for 1h and then declamping.  **Study drug:** Dexmedetomidine (infusion, 2.5/5 micrograms/kg/hr), yohimbine (bolus, 1mg/kg)  **Study duration:** 4 hours | **1.**Dexmedetomidine was associated with a dose dependent improvement in histological evidence of inflammation and oedema. This was also reflected in lung wet/dry ratio.  **2.** Dexmedetomidine was associated with a dose-dependent reduction in TLR4/MyD88 and NFkB activity.  **3.** Dexmedetomidine was associated with a dose-dependent reduction in concentration of IL-6 and TNF-alpha in BAL fluid. | | Pre-treatment with dexmedetomidine was associated with histological evidence of lung injury and circulating cytokines in a rat model of IRI. | |
| **Sugita 2013** | Rats | **Injury:** Renal ischaemia reperfusion injury  **Study drug:** Dexmedetomidine (infusion1/10/20 micrograms/kg/hr), pentobarbital (infusion, 10mg/kg/hr)  The lowest dose of dexmedetomidine infusion was combined with pentobarbital anaesthesia.  **Study duration:** 7 hours | | **1.** Dexmedetomidine was associated with an improved MAP after reperfusion. Bradycardia was noted at the higher dexmedetomidine dose.  **2.**All doses of dexmedetomidine were associated with a reduction in serum creatinine measured after reperfusion.  **3.** Dexmedetomidine was not associated with any change in serum inflammatory marker levels in any dose or drug combination. | | Infusion of dexmedetomidine with a dose from 1 microgram/kg/hr to 20 micrograms/kg/hrwas associated with improvement in cardiovascular status and renal injury in a rat model of IRI. |
| **Uysal 2012** | Rats | **Injury:** IRI via clamping of femoral artery and vein  **Study drug:** Dexmedetomdine (bolus, 10 micrograms/kg, 30 micrograms/kg).  **Study duration:** 12h for samples, 7 days for survival analysis | | **1.**Dexmedetomidine was associated with reduced tissue levels of NO, malondialdehyde, and myeloperoxidase at both 12h and 7 days.  **2.**Epigastric island skin flap necrosis was globally reduced in the groups treated with both doses of dexmedetomidine. | | Bolus dose of either 10 micrograms/kg or 30 micrograms/kg with dexmedetomidine at time of reperfusion may increase survival of a skin flap at 7 days. |
| **Zhang 2017** | Rats | **Injury:** IRI via occlusion of left anterior descending coronary artery.  **Study drug:** Dexmedetomidine (infusion, 0.7 micrograms/kg/hr), yohimbine (0.5 micrograms/kg/hr)  **Study duration:** 3 hours | | **1.** Pre-treatment with dexmedetomidine was associated with significant reduction in myocardial infarct size. Yohimbine co-administration attenuated this reduction.  **2**.Dexmedetomidine was associated with more intact myocardial fibres with less disarrangement. Yohimbine significantly reduced this protective effect.  **3.**Dexmedetomidine pre-treatment was associated with attenuation of all measured inflammatory markers. This effect was inhibited by co-administration of yohimbine. | | Pre-treatment with dexmedetomidine infusion may reduce infarct size and histological evidence of myocardial injury in a rat model of IRI. This effect may be mediated via the alpha-2 receptor. |
