# Supplementary material for: The effect of dexmedetomidine and clonidine on the inflammatory response in critical illness: a systematic review of animal and human studies
Source: Crit Care. 2019 Dec 11;23:402. doi: 10.1186/s13054-019-2690-4 (PMC6907244; doi:10.1186/s13054-019-2690-4)
Supplement: Supplementary file 3 — Additional file 3. Detailed description of animal studies. [file 13054_2019_2690_MOESM3_ESM.doc]

| **Author** | **Population** | **Intervention/Protocol** | **Outcome** | | | **Key Findings** | **Strengths and Weaknesses** | **Main Conclusions** |
| --- | --- | --- | --- | --- | --- | --- | --- | --- |
| **Lung Injury** |  |  |  | | |  |  |  |
| **Chen 2014** | This study was performed in 30 healthy dogs divided into 5 groups of 6.  There was 1 control group, 1 group ventilated only, and 3 other groups ventilated after exposure to varying doses of dexmedetomidine. | In the 4 ventilated groups, ventilator induced lung injury (VILI) was caused by tidal volumes of 20ml/kg at a respiratory rate of 15/minute. Total mechanical ventilation (MV) time was 4 hours.  Each group receiving dexmedetomidine was given an initial bolus (0.5/1.0/2.0 micrograms/kg) prior to ventilation and subsequent infusion (0.5/1.0/2.0 micrograms/kg/hr) started within 20 minutes of intubation. This was continued for 4 hours (h), after which the study period ended and tissue samples were taken. | Lung histopathology  Lung injury score  Lung weight  Tumour necrosis factor-alpha (TNF-alpha), myeloperoxidase (MPO), polymorphonuclear neutrophils (PMNs), nuclear factor-kB (NFkB) messenger RNA, inducible nitric oxide synthase (iNOS) messenger RNA were all measured in lung tissues. | | | Partial pressure of oxygen (PaO2) were significantly decreased in all ventilated groups at 4h of MV (although no statistical difference found between treatment and control groups).  **Lung histopathology**: a dose dependent reduction in intra-alveolar exudates and inflammatory cell infiltration with increasing dexmedetomidine dose.  **Inflammatory markers**: Lung tissue concentration of myeloperoxidase, polymorphonuclear neutrophils, nuclear factor-Kb (NFkB) messenger RNA (mRNA) and inducible nitric oxide synthase (iNOS) mRNA were all significantly reduced in the 1.0mcg/kg/hr and 2.0mcg/kg/hr dexmedetomidine groups. | Dexmedetomidine was administered prior to any injury or inflammatory process  No alpha-2 antagonist was used to clarify anti-inflammatory mechanisms.  No statistical difference found in PaO2 between groups.  The doses used are equivalent to human dosage.  A limited number of significant inflammatory molecules were studied, specific to their presence in lung tissue only. | Pre-medication and infusion with dexmedetomidine at clinically relevant doses can reduce histological evidence of inflammation in the lung after VILI in dogs.  This effect seems to be dose dependent. |
| **Jiang 2014** | This study was performed in 72 healthy Sprague-Dawley rats randomly assigned to 6 groups of 12.  Alongside a sham group and control group, one group was given 2.5mcg/kg/hr dexmedetomidine pre-treatment, one given 5.0mcg/kg/hr dexmedetomidine pre-treatment, one given 5.0mcg/kg/hr dexmedetomidine pre-treatment plus alpha-2 antagonist yohimbine prior to this, and another given just yohimbine pre-treatment. | 1hr after drug pre-treatment, an ischaemia-reperfusion injury (IRI) was caused by occlusion of left pulmonary hilum, bronchus, artery, and vein for 1h.  All samples were taken 2h after reperfusion was started.  All drugs given as 1h infusion prior to IRI. | Histology  Lung weight  Bronchoalveolar lavage (BAL): TNF-alpha, interleukin-6 (IL-6), monocyte chemoattractant protein-1 (MCP-1), MPO  Quantitative polymerase chain reaction(qPCR) from lung tissue– Toll-like Receptor 4 (TLR4), myeloid differentiation primary response 88 (MYD88)  Western Blot - P-JNK, P-ERK | | | **Lung histopathology**: Lung injury score was reduced by dexmedetomidine pre-treatment by reduction in inflammatory cell infiltration seen in lung tissue.  **Inflammatory markers** MPO, TNF-alpha, IL-6, TLR4 and MyD88 were all attenuated by dexmedetomidine at both doses.  P-JNK and P-ERK expression were both reduced by dexmedetomidine in a dose-dependent matter.  Combination with yohimbine only partially reversed the anti-inflammatory effect of dexmedetomidine. | Dexmedetomidine was administered prior to any injury or inflammatory process  5mcg/kg/hr is higher than equivalent human dosage. | Pre-treatment with dexmedetomidine is associated with attenuation of pulmonary damage caused by IRI in rats.  This effect was not consistently dose dependent.  This effect was only partially reversed by Yohimbine, suggesting an alternate mechanism for dexmedetomidine’s anti-inflammatory effect. |
| **Loftus 2017** | 80 Sprague Dawley rats randomly assigned to 10 study groups of 6-8.  1 Control  2 Lung contusion (LC)  3 LC + Clonidine (CLO)  4 LC + Propranolol (PPL)  5 LC + Haemorrhagic shock (HS)  6 LC + HS + CLO  7 LC + HS + PPL  8 LC + HS + Daily Restraint Stress (DRS)  9 LC + HS + DRS + CLO  10 LC + HS + DRS + PPL | 10 minutes after traumatic insult, the rats were given an intraperitoneal injection of clonidine (75mcg/kg), or propranolol (10mg/kg) each day for 7 days.  Blood and tissue samples were then taken on day 7 immediately after animal sacrifice by cardiac puncture. | Histology and lung injury score  PCR- TNF-alpha, high mobility group box (HMGB1), vascular endothelial growth factor (VEGF), vascular endothelial growth factor receptor 1 (VEGFR1), vascular endothelial growth factor receptor 2 (VEGFR2) | | | **Lung histopathology**: Daily clonidine administration was associated with increased VEGF and VEGF receptor expression. This was associated with reduced histological inflammation and lung injury score  Propranolol had a similar, but less pronounced and less consistent anti-inflammatory effect.  **Inflammatory markers:** TNF-alpha was reduced by both study medications, but more so by clonidine. | Both substances administered after inflammatory insult, and given daily along with chronic inflammatory insult, which may be more applicable to a clinical scenario.  Comparison of beta blockade with alpha-2 agonist elucidates more mechanistic information, suggesting that clonidine’s central effects (versus propranolol’s peripheral effects) are more important in upstream control of the inflammatory pathway.  The dose of clonidine is far higher than we would use clinically. | In high doses, administered regularly after daily traumatic insult, clonidine can increase VEGF and its receptor expression in rat lungs. This effect is more pronounced with clonidine in comparison to propranolol. |
| **Yang 2008** | 64 healthy rats were randomised to 4 study groups of 16.  The first group was given ‘standard ventilation’ at 10ml/kg tidal volume, while the remaining three were ventilated at 20ml/kg to induce lung injury.  Of the lung injury groups, one was given no additional intervention, one given dexmedetomidine, and one given dexmedetomidine plus yohimbine (alpha-2 antagonist) | After 150 minutes of ventilation, two of the groups were given a 1 microgram/kg loading dose of dexmedetomidine, prior to infusion at either 0.5 ,2.5, or 5 micrograms/kg/hr. One of these groups was then also given a bolus of 0.1mg/kg yohimbine immediately after dexmedetomidine bolus.  The study was continued for 5 hours at which point samples were taken at animal sacrifice. | Lung histology  Arterial blood gases  TNF-alpha, IL-6, interleukin-1b (IL-1b) iNOS, cyclo-oxygenase 2 (COX-2), B-actin | | | Dexmedetomidine infusion at 5 micrograms/kg/hr attenuated lung injury score, inflammatory infiltration on lung histology, and expression of inflammatory molecules. This was not seen with any lower dose. | All study drugs were given a significant period of time after lung injury was induced, making this more applicable to a clinical scenario.  Alpha-2 antagonist yohimbine was used in order to elucidate the role of the alpha-2 receptor in the study’s findings. | Infusion of dexmedetomidine at 5 micrograms/kg/hr may br associated with reduced lung injury score and inflammatory markers.  There was significant (but not complete) attenuation of this anti-inflammatory effect when combined with yohimbine. This suggests a crucial role for the alpha-2 receptor in the inflammatory pathway. |
| **Yang 2011** | 84 healthy rats were randomised to 7 groups of 12.  In addition to saline only, VILI only, LPS only, and LPS with VILI groups, each model of injury (solo and combined) were also treated with dexmedetomidine and ketamine combination therapy. | VILI was induced by 20ml/kg ventilation  Endotoxaemia was induced by LPS administration  Immediately after the injury was caused, 1mcg/kg bolus and 0.5mcg/kg/hr infusion of dexmedetomidine was started, alongside an infusion of 1mg/kg/hr ketamine.  The study lasted 4 hours, after which samples were taken at animal sacrifice. | Lung histology and injury score  NO, IL-1b, COX2, prostaglandin E2 (PGE2), macrophage inflammatory protein 2 (MIP2), iNOS mRNA | | | **Lung histopathology:** Groups which received dexmedetomidine and ketamine had reduced inflammatory infiltration on lung histology.  **Inflammatory markers**: Groups which received dexmedetomidine and ketamine showed reduced levels of all circulating inflammatory molecules.  There was no difference seen between VILI, LPS, or VILI + LPS groups. | The study subjects were only given dexmedetomidine and ketamine in combination, with no individual effect of each studied  The group with endotoxaemia and VILI was thought to replicate patients with severe sepsis who are mechanically ventilated with evidence of sepsis-induced lung injury.  Both study substances were given at doses within the clinical range.  Clinically, it would be rare to use both ketamine and dexmedetomidine in combination as sedatives in the ICU.  Previous data shows that both agents may only have anti-inflammatory effects at very high dosages. This study suggests combination at lower, clinical doses can produce an anti-inflammatory effect. | The combination of dexmedetomidine and ketamine in clinically relevant doses may have an anti-inflammatory effect in models of VILI and endotoxaemia. |
| **LPS** |  |  |  | | |  |  |  |
| **Chen 2015** | This study involved 40 healthy rats randomised into 5 groups of 8.  In addition to a control group, and a group given only E. Coli lipo-polysaccharide (LPS), one group was given LPS and dexmedetomidine, another given LPS and yohimbine (alpha-2 antagonist), and the fifth group given LPS, dexmedetomidine and yohimbine. | Endotoxaemia was induced by LPS injection.  The treatment groups were given dexmedetomidine at 5 micrograms/kg/hr 30 mins after LPS, yohimbine at 250 micrograms/kg (bolus) immediately prior to LPS injection, or both.  The rats were studied for a total of 6 hours. Blood samples were taken at this time from the abdominal aorta immediately after animal sacrifice. | Endotoxaemia-induced hepatic injury was the primary outcome assessed.  Serum aspartate aminotransferase (AST), alanine aminotransferase (ALT), and lactate were measured.  Liver tissue was then analysed for TNF-alpha, IL-6, and IL-1b along with measures of liver cell apoptosis.  Degree of liver tissue oxidative stress was measured using malondialdehyde (MDA) and superoxide dismutase (SOD). | | | **Liver histology**: Dexmedetomidine at 5 micrograms/kg/hr significantly reduced levels of liver cell apoptosis, cytokine concentration, and markers of oxidative stress.  **Serum markers:** AST and ALT were reduced by 30% with dexmedetomidine administration, along with lactate levels.  Yohimbine co-administration completely reversed any beneficial effect of dexmedetomidine on liver function tests, inflammation, and histological appearance. | Dexmedetomidine was administered at a concentration around 10 times higher than a clinically relevant dose for a human. However, more realistically, it was administered after the ‘septic injury’ had begun.  This study looked specifically at endotoxaemia-induced liver injury as an outcome, as opposed to any systemic marker of improvement. Dexmedetomidine may be improving liver injury due to systemic anti-inflammatory effects, reducing the cytokine and oxidative stress ‘hit’ on the liver. However, this can only be hypothesised from this study’s results.  Yohimbine co-administration completely abolished any beneficial effect of dexmedetomidine. | Dexmedetomidine at 5 micrograms/kg/hr improves liver injury score after endotoxaemia is induced by LPS in rats. This effect is completely reversed by administration of an alpha-2 antagonist. |
| **Feng 2019** | This study involved 42 adult male rats divided into 7 equal groups.  The control group received saline as an intraperitoneal injection. One group was given LPS ony, one group was given DEX at 25micrograms/kg via intraperitoneal injection, one group was given LPS+ GSK-3b, one group was given Atipamezole (APZ)+DEX+LPS, one group was given Idazoxan IDA+DEX+LPS and the last group vehicle+LPS. | In relevant groups DEX was administered at 25micrograms/kg as a bolus 30min prior to LPS.  ATZ was given at 250micrograms/kg as a bolus 30min prior to LPS  GSK-3b was given at 10mg/kg 30min prior to LPS  The study lasted for 4 hours at which point serum , tissue and urine samples were taken. | Renal function  Histological appearance  Inflammatory cytokines: IL-1b, TNFa, IL-6, IL-8  Oxidative stress markers: ROS, MDA, GSH  Expression of p-Ser9-GSK-3b and Nrf2 | | | **Renal Function:** DEX attenuates LPS induced renal function. This effect was attenuated by ATZ and IDA  **Histology:** DEX attenuates LPS induced renal structural damage. ATZ and IDA reversed this effect.  **Inflammatory cytokines:** DEX attenuated the LPD induced rise in cytokine level. This effect was reversed in APZ and IDA.  **Oxidative stress:** DEX attenuates LPS induced oxidative stress. The effect was reversed with APZ and IDA.  DEX promotes Ser9 phosphorylation of GSK-3b and accumulation of Nrf2 in the nucleus during LPS induced AKI. | DEX given as an intraperitoneal bolus and low dose.  APZ and IDA, alpha-2 adrenoreceptor antagonists reversed the attenuating effects of DEX. | DEX attenuated pro-inflammatory cytokines induced by LPS. This effect was reversed by alpha-2 antagonists. |
| **Kang 2018** | This study involved 60 male mice that were divided equally into 5 groups.  The control group received saline. One group received only LPS, one group was given ABT (1 microgram/kg)+ LPS, one group was given DEX (40 micrograms/kg)+ LPS and one group had DEX, ABT and LPS. | AKI was induced by LPS injection.  DEX (40micrograms/kg) and ABT (1microgram/kg) were administered by bolus one hour pre-LPD intraperitoneally.  After 0, 9, 16 and 20 hours kidney tissue samples were extracted.  The study lasted 24 hours | Serum TNF alpha and IL-6  Kidney tissue histology | | | **Renal histology:** DEX attenuates LPS induced renal pathological injury. These effects were not seen when administered with ABT.  **Inflammatory cytokines:** DEX attenuates the LPS induced rise in serum inflammatory cytokines. This effect was revered by ABT. | Intraperitoneal bolus rather than infusion was used.  The attenuation of renal damage and inflammatory cytokines was reversed when administered with an alpha-2 antagonist. | Dexmedetomidine as a pre-LPS intraperitoneal bolus attenuates renal pathology serum inflammatory cytokines.  This effect is attenuated by ABT. |
| **Kong 2017** | This study involved randomisation of mice to 5 study groups (unknown group size). Alongside a control group and a group administered with only LPS, one group was given dexmedetomidine at 10mg/kg alongside LPS, one given alpha-bungarotoxin (ABT*) at 1 microgram/kg alongside LPS, and one given both study drugs as well as LPS.  *ABT is an antagonist at the alpha-7 nicotinic acetylcholine receptor present in the central nervous system. | Septic cardiomyopathy was induced by injection of LPS.  Dexmedetomidine groups were given the study drug (bolus) 1 hour before LPS.  ABT groups were given this (bolus) 1 hour before LPS, and 1 hour before dexmedetomidine.  Mice were initially treated for varying study lengths to determine optimum histological evidence of septic cardiomyopathy. This was decided to be 16 hours study length. | Myocardial tissues were analysed for concentrations of IL-6, IL-1b, and TNF-alpha and histological appearance.  Pro-inflammatory intracellular messengers such as NF-Kb and p-STAT3 were also assessed.  Alpha-7 nicotinic acetylcholine receptor levels were measured in myocardial tissue  Pro-apoptosis proteins caspase-3, caspase-8, BCL2, Bax, and p53 were all measured. | | | **Myocardial histology:** Treatment with dexmedetomidine markedly reduced myocardial tissue damage and apoptosis. This improvement was abolished when co-administered with ABT.  Administration of dexmedetomidine reduced all pro-apoptotic protein levels, alongside tissue concentrations of all cytokines, along NF-Kb.  **Cholinergic pathway:** Alpha-7 nicotinic acetylcholine receptor was decreased as a result of endotoxaemia, but increased when dexmedetomidine was administered. Administration of its antagonist (ABT) abolished the protective effects of dexmedetomidine. Myocardial apoptosis and all cytokines returned to pre-dexmedetomidine levels. | This study involved an incredibly large dose of dexmedetomidine (10mg/kg) as single bolus prior to septic injury.  All outcomes involved only measurements of single organ injury (myocardium).  Administration of ABT suggests that the protective effects of myocardium may be due to dexmedetomidine’s action of the alpha 7 nicotinic acetylcholine receptor. This gives further information into the anti-inflammatory mechanism of alpha-2 antagonists.  A bolus dose of dexmedetomidine does not achieve a steady state serum concentration in the same way an infusion can. | Dexmedetomidine at 10mg/kg (bolus) is associated with reduced myocardial apoptosis and inflammation after endotoxaemia-induced septic cardiomyopathy in mice.  Its potentially protective effects on the myocardium may be through its action in the cholinergic pathway via alpha 7 nicotinic acetylcholine receptor. |
| **Miranda 2015** | 49 Golden Syrian hamsters were randomised to 5 different study groups. Alongside a control group, and a saline administration group, one group was given dexmedetomidine only at 5 micrograms/kg/hr, one given LPS with saline, and one given LPS with dexmedetomidine at 5 micrograms/kg/hr. | In relevant groups, dexmedetomidine was administered as a 3 hour infusion, 1 hour after injection of LPS.  The study lasted 4 hours in total. During this time, the animals’ mean arterial pressure (MAP), heart rate (HR) and temperature were measured. Microvascular function was also measured using intravital microscopy. Fluorescent microscopy was used to assess venular leukocyte-endothelial interactions. | Mean arterial pressure, heart rate, and temperature.  Arterial blood gas and lactate measurements were made.  Microcirculatory changes were measured via intravital microscopy. Fluorescent microscopy was used to determine leukocyte-endothelial interactions. | | | **Microvascular function:** Induction of endotoxaemia by LPS caused increased venular leukocyte rolling and adhesion, reduced functional capillary density, and reduced erythrocyte velocity. Dexmedetomidine treatment significantly attenuated this response.  **Cardiovascular physiology:** Dexmedetomidine treatment caused an expected reduction in heart rate (more so in the endotoxaemic group) but this did not cause a reduction in MAP.  Dexmedetomidine caused a statistically significant reduction in lactate in the endotoxaemic groups. | This study used a large dose of dexmedetomidine, given 1 hr after induction of endotoxaemia.  Measurement of skin and subcutaneous microcirculation may not be representative of solid organ changes.  No serum inflammatory markers were measured, so no correlations can be made between leukocyte-endothelial interactions and inflammatory response.  No fluid resuscitation or norepinephrine was used in the sepsis groups. | Dexmedetomidine treatment at 5 micrograms/kg/hr was associated with improved microcirculatory function in a hamster model of endotoxaemia. |
| **Sezer 2010** | 40 Wistar rats were randomised into 4 groups of 10.  One group was given simply intravenous saline, and one given only LPS. A third group was given dexmedetomidine infused at 5 micrograms/kg/hr only, and a fourth group given LPS and dexmedetomidine infusion (at same rate). | In the relevant groups, dexmedetomidine was administered immediately after injection of LPS.  The study ran for 8 hours, after which the animals were sacrificed and midline laparotomy was performed. | Histological appearance of the liver was examined taking note of central venous congestion, dilation of hepatic sinusoids, and inflammation of the portal tracts. | | | After conjugate scoring for markers of hepatic injury, all groups showed a statistically significant difference between one another.  Dexmedetomidine reduced histological appearance of hepatic injury in endotoxaemia. | This study used an infusion of 5 micrograms/kg/hr of dexmedetomidine immediately after induction of endotoxaemia.  Only specific characteristics of gross liver histology were measured – not including inflammatory cell infiltration or hepatic apoptosis.  Investigators found histological evidence of inflammation even in the control groups where no LPS was administered. | An infusion of dexmedetomidine at 5 micrograms/kg/hr may reduce the histological evidence of hepatic injury in a rat model of endotoxaemia. |
| **Shi 2012** | 40 Sprague Dawley rats were randomised into 5 groups of 8.  Alongside a control group (saline) and an LPS only group, the remaining groups were giving LPS and dexmedetomidine at either 0.5 micrograms/kg, 1.5 micrograms/kg, or 4.5 micrograms/kg | After induction of presumed acute lung injury (ALI) by injection of 4mg/kg LPS, the relevant groups were immediately given the varying dose boluses of dexmedetomidine.  The study lasted 6 hours. | Lung wet/dry ratio was measured to determine presence of pulmonary oedema.  Lung histology was assessed for inflammatory cell infiltration and parenchymal appearance.  Lung tissue NF-Kb was measured using immunohistochemistry  Lung tissue TNF-alpha, IL-1b, and IL-6 were measured using ELISA.  Lung tissue TLR4 mRNA was measured. | | | **Wet/dry ratio:** The wet/dry ratio of the lungs was reduced in the ALI groups with the 1.5 and 4.5 microgram/kg doses of dexmedetomidine. This reduction was not seen with 0.5 micrograms/kg.  **Lung histology:** This trend was replicated in the histological appearance of the lungs. The moderate and high dose dexmedetomidine groups showed reduced inflammatory cell infiltration, congestion, and alveolar wall thickening.  **Inflammatory pathways:** The moderate and high dose dexmedetomidine groups also showed a reduction in NF-Kb expression. This was the same for TLR4 mRNA expression and cytokine concentration. | Acute lung injury (ALI) induced by LPS is not immediately translatable to ALI in a critically unwell, possibly ventilated, human.  Dexmedetomidine was administered immediately after LPS.  The dose-dependent findings are consistent with many previous animal studies.  A bolus regime instead of infusion means that a stable blood concentration of dexmedetomidine will not have been achieved.  Cardiovascular physiology was not monitored during the experiment, so the safety of the higher doses of dexmedetomidine cannot be validated. | Dexmedetomidine at 1.5 micrograms/kg and 4.5 micrograms/kg bolus is associated with reduced evidence of ALI induced by LPS in a dose-dependent manner. |
| **Szelenyi 2000** | 60 mice were randomised into 6 groups of 8-10.  1 Control  **Alpha-2 agonist groups**  2 UK 14304 (5mg/kg)  3 Xylazine (5mg/kg)  4 Clonidine (5mg/kg)  **Alpha-2 antagonist groups**  5 CH- 38083 (10mg/kg)  6 WB-4101 (10mg/kg)  7 Prazosin (5mg/kg) | Alpha-2 agonist treatment groups were administered the study drug via intraperitoneal bolus 30 minutes prior to LPS.  Alpha-2 antagonist treatment groups were administered the study drug via intraperitoneal bolus 60 minutes prior to LPS.  The study lasted 90 minutes, after which the animals were sacrificed and blood samples taken. | IL-10 | | | All alpha-2 agonists used caused a significant reduction in IL-10 measurements in comparison to LPS only.  Alpha-2 antagonists caused an increase in IL-10 in comparison to LPS only | Significantly large doses of clonidine were used in this study.  All drugs were given as an intraperitoneal bolus prior to LPS.  No cardiovascular monitoring or measurement of mortality was made during the study.  Only one blood sample was taken after only 90 minutes of LPS. | Alpha-2 agonists may be associated with reduced IL-10 levels at 90 minutes after LPS administration in mice.  This effect may be due to alpha-2 receptor activity, as it is almost entirely reversed by the administration of alpha-2 antagonists. |
| **Tan 2015** | 32 rats were randomised into 4 groups of 8.  Alongside a control group and LPS only group, a group was given LPS and dexmedetomidine at 10 micrograms/kg, and a final group was given LPS with dexmedetomidine and yohimbine (alpha-2 antagonist) at 1mg/kg. | The bolus of dexmedetomidine was given immediately prior to LPS administration. The yohimbine group received this 30 minutes prior to dexmedetomidine.  The study lasted 4 hours, after which the animals were sacrificed, blood samples taken from the abdominal aorta, and renal tissue taken for histological analysis. | Serum creatinine was measured to determine presence of endotoxaemia-induced acute kidney injury (AKI)  Plasma IL-6, IL-18, and TNF-alpha were measured to determine systemic cytokine load.  Western blot analysis was performed on renal tissue to detect presence of kidney injury molecule 1 (KIM-1), and high mobility group protein 1 (HMGB-1) | | | **Acute Kidney Injury:** Endotoxaemia with LPS doubled the serum creatinine in the animals. Pre-treatment with dexmedetomidine was protective of the kidneys, causing normal creatinine levels during endotoxaemia. The presence of yohimbine removed this protective effect.    **Renal histology:** Dexmedetomidine decreased visible renal tubular epithelial cell degeneration and inflammatory cell infiltration. Combination with yohimbine showed a return of renal tissue damage and inflammation. This same pattern was shown in the expression of KIM-1 and HMGB-1.  **Inflammatory markers:** All inflammatory markers were raised in endotoxaemic rats. Their levels were significantly reduced by pre-treatment with dexmedetomidine. This anti-inflammatory effect was abolished in groups treated with dexmedetomidine and yohimbine. | This study involved a pre-treatment of a very large dose of dexmedetomidine.  KIM-1 and HMGB-1 are both known to have a role in the inflammatory cascade. Their measured expression profile here is suggestive that AKI may be due to the inflammatory effect of LPS, and that dexmedetomidine’s renal protection may be due to an anti-inflammatory mechanism.  The use of yohimbine showed that both the renal protective and anti-inflammatory effect of dexmedetomidine are very likely alpha-2 receptor mediated, and linked.  No cardiovascular physiology was measured during the study period, so the potential detrimental effects of this high dexmedetomidine dose cannot be excluded. | Pre-treatment with a bolus of 10 micrograms/kg of dexmedetomidine can reduced renal injury in a rat model of LPS-induced endotoxaemia. This may be associated with its anti-inflammatory profile, mediated via the alpha-2 receptor. |
| **Taniguchi 2004** | 57 rats were randomised into 4 groups of 10-16.  In addition to a control group, an LPS only group, and a dexmedetomidine (5 micrograms/kg/hr) group, the final group received LPS and dexmedetomidine. | In the LPS/dexmedetomidine group, dexmedetomidine was given as infusion at 5 micrograms/kg/hr started immediately after LPS administration.  The animals were monitored for cardiovascular parameters and mortality for 8 hours, at which point the study was stopped.  Blood samples were taken at 1h, 3h, and 5h.  Lung tissue was resected at necropsy to assess histology. | The animals had MAP and HR measured during the study along with regular arterial blood gases.  Plasma TNF-alpha and IL-6 levels were measured at regular intervals.  Lung histology was assessed for any signs of inflammation, oedema, or parenchymal injury.  Mortality was assessed at 8 hours for all study groups. | | | **Mortality:** 8h mortality was 94% for the LPS only group. This was reduced to 44% when also treated with dexmedetomidine infusion.  Dexmedetomidine alone showed 0% mortality.  **Cardiovascular physiology:** The LPS only group became expectedly significantly hypotensive. This was returned to almost-normal blood pressures when also given dexmedetomidine.  **Inflammatory markers:** Plasma TNF-alpha and IL-6 were both reduced by dexmedetomidine during endotoxaemia. This reduction was more marked with increasing time during the study.  **Lung histology:** Dexmedetomidine treatment reduced gross signs of lung injury and inflammatory cell infiltration during endotoxaemia. | This study used a relatively large dose of dexmedetomidine as infusion immediately after inflammatory insult.  Measurement of cardiovascular parameters and mortality gives more clinically relevant information for the use of dexmedetomidine in sepsis/inflammatory conditions.  Regular measurement of plasma cytokine concentration shows that the beneficial effects of dexmedetomidine may be time-dependent. | Dexmedetomidine infusion at 5 micrograms/kg/hr is associated with reduced global markers of inflammation including plasma cytokines and lung inflammation, in a rat model of LPS-induced endotoxaemia. This was also associated with improved blood pressure profile and reduced mortality. |
| **Taniguchi 2008** | 96 healthy Wistar rats were randomised into 7 groups of 12-15.  4 of these groups were designed to study **dose-related** effects of dexmedetomidine:  1 LPS  2 LPS + DEX (2.5mcg)  3 LPS + DEX (5mcg)  4 LPS + DEX (10mcg)  3 groups were designed to study **time-related** effects of dexmedetomidine:  1 LPS  2 LPS + DEX (10mcg) 1h afterwards  3 LPS + DEX (10mcg) 2h afterwards | **Dose-related effects**  Infusion of dexmedetomidine at 3 different rates (in mcg/kg/hr) were started in three different groups immediately prior to administration of LPS.  Arterial blood gases were taken at 1, 3, and 5h.  Plasma cytokine levels were taken at 2, 4, and 5h.  **Time-related effects**  The study animals were injected with LPS and then given an infusion of 10mcg/kg/hr dexmedetomidine either 1h or 2h after LPS administration.  Blood samples were taken using the same protocol as the drug-related effect study groups.  All studies lasted 8h in total. | Cardiovascular parameters including systolic arterial pressure (SAP) and HR were measured throughout the study. Mortality rates were measured up until 8h.  Plasma TNF-alpha and IL-6 were measured at 2, 4, and 5h time points. | | | **Cardiovascular physiology:**  The LPS-only groups experienced significant hypotension. All groups given dexmedetomidine had no hypotension, with exception of the late post-treatment group (2h post LPS). No study groups experienced bradycardia.  **Mortality:** All groups that received dexmedetomidine had significantly lower mortality rates than those which did not. This benefit was the most modest in the group receiving dexmedetomidine 2h after LPS.  **Inflammatory markers:** Plasma TNF-alpha and IL-6 were reduced in all groups that received dexmedetomidine. This was dose-dependent, with the least significant reduction in the group receiving 2.5mcg/kg/hr. The group receiving dexmedetomidine 2h after LPS also showed only a modest decrease in both inflammatory markers. | This study helps to add more detail to the dose-related and time-related effects of dexmedetomidine.  The dose-dependent decrease in inflammatory markers suggests one of its mechanisms of action may be modulation of cytokine production by monocytes and macrophages.  The improved mortality with only the smallest dose of dexmedetomidine alongside its protective effects on haemodynamics may also suggest this may also play a role in its beneficial profile.  The dose of LPS used in this study was particularly high, and produced a high mortality rate. This was a conscious decision to allow the beneficial effects of dexmedetomidine to be elucidated. | In a model of LPS-induced endotoxaemia in rats, dexmedetomidine pre-treatment shows a safe cardiovascular profile, with a dose-dependent reduction in plasma inflammatory markers and mortality.  Delayed administration of dexmedetomidine after an inflammatory insult (2h) produces only modest benefit in comparison to early administration (1h). |
| **Wu 2014** | 30 mice were randomised into 5 groups of 6.  In addition to a control, and LPS only group, one group was given dexmedetomidine only at 50mcg/kg/hr, another was given the same dose along with LPS, and a final group was given a reduced dose at 5mcg/kg/hr along with LPS. | Dexmedetomidine infusions were started immediately after injection of LPS.  This study lasted 8 hours after which it is assumed whole blood samples were taken.  Peritoneal sampling was taken to represent white cell number and activity at the original ‘infection site’ where LPS was introduced. | Whole blood and peritoneal samples were analysed for lymphocyte proliferation, macrophage activity, and NK cell activity. | | | Low dose dexmedetomidine reduced numbers of peripheral T cells and B cells in whole blood, blunting the increase caused by LPS. It had no effect on peripheral numbers of NK cells, macrophages, and monocytes.  High dose dexmedetomidine had no further effect on peripheral T cell and B cell numbers, but did decrease numbers of macrophages seen.  Dexmedetomidine reduced systemic macrophage phagocytosis at low dose and high dose to the same degree.  Dexmedetomidine increased systemic NK cell activity, but even more so at the higher dose.  ‘Infection site’ samples showed preserved macrophage activity at low dose, and enhanced it at high dose. | This study helpfully provides further information on the effects of dexmedetomidine at different doses in the adaptive and innate immune system.  Both systemic and ‘infection site’ sampling allowed a more detailed profile of activity to be assessed, although an LPS model has limits to its ability to replicate true site-specific infection.  The study itself does not specifically state the time at which the cell samples were taken from the study subjects.  No comments are made on the mortality and cardiovascular status of the mice throughout the study. | In a mouse model of LPS-induced endotoxaemia, 5 mcg/kg/hr infusion of dexmedetomidine is associated with decreased peripheral B cell and T cell augmentation, while preserving ‘infection site’ macrophage and systemic NK cell activity.  High dose dexmedetomidine seemed to enhance NK cell and ‘infection site’ macrophage activity. |
| **Xiang 2014** | 168 rats were randomised into 2 groups of 20, and 8 groups of 16.  **Survival analysis**  1 LPS + Saline  2 LPS + DEX  **Alpha-bungarotoxin (ABT)**  1 LPS + Saline  2 LPS + DEX  3 LPS + ABT + DEX  4 LPS + Saline + DEX  **Vagotomy (VNX)**  1 LPS + VNX + DEX  2 LPS + Sham + DEX  **Vagal activity analysis**  1 Saline  2 DEX | In relevant groups, dexmedetomidine was given as a bolus of 40 micrograms/kg  In relevant groups, alpha-bungarotoxin (alpha 7 nicotinic acetylcholine receptor antagonist) was given as a bolus of 1 microgram/kg.  These were both given at least 15 minutes prior to injection of LPS.  For the survival analysis, the animals were studies for a total of 120h. All other studies lasted 3h, at which point plasma cytokine samples were taken via cardiac puncture.  The cervical vagus nerve was used for VNX and activity analysis. | Endpoints studied were animal survival, plasma concentrations of TNF-alpha, IL-1b, and IL-6, and vagal nerve activity. | | | **Survival analysis:** The group receiving dexmedetomidine pre-treatment survived for significantly longer than their control counterparts. A 120h, this was statistically significant at a survival rate of 0.65 vs 0.25 in the saline group.  **Inflammatory markers:** All studied inflammatory markers showed a significant reduction when measured at 3h when dexmedetomidine pre-treatment was given. This effect was abrogated by further pre-treatment with ABT.  **Vagotomy:** The anti-inflammatory effect of dexmedetomidine pre-treatment was completely abolished in the mouse model of vagotomy.  **Vagal activity:** Dexmedetomidine significantly increased vagal nerve discharge frequency (346 Hz vs 179Hz in saline group), but had no effect on discharge amplitude. | This study provides a structure analysis of the anti-inflammatory effect of dexmedetomidine via the cholinergic pathway.  It uses only a single bolus of a very high dose (40 micrograms/kg) in place of infusion. Only one dose was used, with no ability to determine steady state plasma concentration.  Survival analysis lasted for 120h, giving a more thorough assessment of mortality on a longer term. However, survival was not measured with co-administration of ABT or with vagotomy.  Cardiovascular status was not monitored during the experiments.  Use of ABT and VNX show there is convincing evidence for dexmedetomidine’s mechanisms via the cholinergic pathway. | Dexmedetomidine as a pre-treatment bolus of 40 micrograms/kg is associated with increased survival and reduced plasma inflammatory markers in a rat model of LPS-induced endotoxaemia.  This anti-inflammatory effect is abrogated with both vagotomy and alpha-bungarotoxin. This suggests a role for the cholinergic pathway in the anti-inflammatory mechanism of dexmedetomidine. |
| **Yeh 2016** | 92 rats were randomised into 4 groups of 23.  In addition to a control group, an LPS only group, and a dexmedetomidine only group, the final group was given both LPS and dexmedetomidine. | Dexmedetomidine was given as an infusion at 5 micrograms/kg/hr and started at the same time as LPS was given.  Blood samples were taken at 0, 30mins, 60 mins, 120 mins, and 240 mins. The study lasted 240 mins in total. | Cardiovascular parameters were assessed throughout the study.  Serum Endocan level  Small bowel microcirculatory parameters | | | **Cardiovascular physiology:** There was no significant difference in blood pressure or heart rate when dexmedetomidine was added to the rat model of LPS.  **Small bowel microcirculation:** Dexmedetomidine infusion improved microcirculatory flow, reduced endothelial dysfunction, attenuated intestinal epithelial cell death, and reduced intestinal bacterial translocation in an LPS model.  **Serum Endocan:** This was reduced when dexmedetomidine was combined with LPS. This suggests it is protective against endothelial dysfunction. | Analysis of cardiovascular status for the 4 hour study length shows that 5 micrograms/kg/hr of dexmedetomidine has no detrimental effect on heart rate or blood pressure in an LPS model.  The investigation of microcirculation was limited to only the terminal ileum, and no other locus or organ.  No central venous or arterial blood gases were taken throughout the study period. | Infusion of dexmedetomidine at 5 micrograms/kg/hr in a rat model of LPS showed no harmful effect on cardiovascular status. It was associated with improvements in small bowel microcirculation and reduced markers of endothelial dysfunction. |
| **CLP** |  |  |  | | |  |  |  |
| **Chen 2015** | 64 Sprague Dawley rats were randomised to 4 groups of 16.  In addition to a sham and caecal ligation and puncture (CLP) only group, another group was given CLP and dexmedetomidine (bolus 5 micrograms/kg), and a fourth group was given CLP, dexmedetomidine, and yohimbine (bolus 1mg/kg) | Dexmedetomidine was infused over 1 hr, and given 30 minutes after CLP.  Yohimbine was infused over 15 minutes, and given immediately prior to dexmedetomidine in the combination group.  Serum and intestinal tissue samples were taken at 12h and 24h into the study.  10 animals from each group underwent survival analysis over a 7-day period. | 7 day mortality  Intestinal histology  Serum levels of diamine oxidase, D-lactate, TNF-alpha, IL-1b, and IL-6 were assessed at 12 h and 24 h.  Intestinal TLR4 was also measured at 12h and 24h. | | | **Mortality:** Dexmedetomidine reduced 7-day mortality from 80% (CLP only) to 30% (CLP and dexmedetomidine). Addition of its alpha-2 antagonist yohimbine (CLP, dexmedetomidine, and yohimbine) increased mortality almost back to original rates (70%).  **Intestinal histology:** Dexmedetomidine ameliorated the intestinal damage caused by CLP. Specifically, it showed improved vili height, and reduced neutrophil infiltration.  **Serum inflammatory markers:** Dexmedetomidine reduced concentrations of all serum inflammatory markers measured. This effect was antagonised by yohimbine.  **Intestinal TLR4:** Dexmedetomidine treatment decreased the TLR4 expression seen in CLP treatment. Treatment with yohimbine attenuated, but did not completely abolish, this effect. | This study used a bolus of 5 micrograms/kg of dexmedetomidine given 30 minutes after intestinal injury.  Both histological and biochemical markers of intestinal injury were used.  A single bolus of dexmedetomidine does not allow for achievement of steady state serum concentration. Serum concentration was also not monitored.  The animals’ cardiovascular parameters were not measured throughout the study.  Only the short term trajectory (up to 24 h) of inflammatory markers was measured. | A single bolus of 5 micrograms/kg of dexmedetomidine may be associated with reduced serum inflammatory markers, intestinal TLR4 expression, and histological evidence of intestinal damage in a rat model of CLP. |
| **Hofer 2009** | 200 rats were randomised into 6 groups.  In addition to three control groups, one group was given clonidine 5 micrograms/kg both 12h and 1h before CLP, as well as 1h, 6h, and 12h after CLP. Another group underwent the same protocol but with dexmedetomidine at 40 micrograms/kg. A final group was given clonidine 5 micrograms/kg only at 1h, 6h, and 12h after CLP. | Serum samples were taken for cytokine analysis at 24 hours.  Cardiovascular parameters were measured throughout the study.  The study ran for 5 days. | 5-day mortality  Cardiovascular physiology  Serum TNF-alpha, IL-6, IL-1b, and NFkB. | | | Pre-treatment with clonidine and dexmedetomidine showed a statistically significant improvement in survival at 5 days, in comparison to CLP only.  Post-CLP treatment only with clonidine showed a trend towards survival, but did not reach statistical significance.  Pre-treatment with clonidine showed improved blood pressure profile in comparison to the hypotension seen in CLP only  Pre-treatment with clonidine reduced serum levels of all cytokines measured. Post-treatment clonidine only showed a much more modest reduction.  NFkB binding activity was reduced by the pre-treatment of clonidine. | This study used a bolus of 5 micrograms/kg of clonidine at regular intervals both before and after CLP.  Ketamine (a non-competitive inhibitor of the nicotinic acetylcholine receptor) was used for anaesthesia in these rats.  The combination of both pre- and post- CLP administration was useful in determining most effective stage at which clonidine has its anti-inflammatory action.  All drug doses were given intra-peritoneally, as opposed to intravenously. | A bolus of 5 micrograms/kg given at regular intervals both pre and post CLP can reduce mortality, improve blood pressure, and reduce serum inflammatory markers in a rat model.  This effect was greater than administration of clonidine at the same dose only after CLP. |
| **Koca 2013** | 21 rats were randomised into 3 groups of 7.  In addition to a sham and CLP only group, a third group underwent CLP and administration of a dexmedetomidine bolus 50 micrograms/kg | Dexmedetomidine was administered intraperitoneally immediately after CLP  The study lasted for 6h after which lung and kidney samples were taken for histology along with blood samples for markers of kidney injury. | Lung histology  Kidney histology  Serum creatinine  Plasma neutrophil gelatinase-associated lipocalin (NGAL)  Kidney tissue malondialdehyde (MDA)  Caspase cleaved cytokeratin 18 (CK18) | | | Dexmedetomidine treatment attenuated all histological markers of both kidney and lung injury.  There was a statistically significant lowering of all biochemical markers of kidney injury when dexmedetomidine was added to rats undergoing CLP. | A large bolus of 50 micrograms/kg of dexmedetomidine was used immediately after CLP.  Both renal and lung histology were analysed.  Highly sensitive biochemical markers of renal injury were used. | A bolus of 50 micrograms/kg of dexmedetomidine immediately after CLP may reduce histological evidence of renal and lung injury alongside biochemical markers of kidney injury. |
| **Qiao 2009** | 60 Sprague Dawley rats were randomised into 3 groups of 20.  In addition to a CLP only group, a second group was given an infusion of midazolam at 0.6mg/kg/hr, and a third group was given CLP and infusion of dexmedetomidine at 5 micrograms/kg/hr | The infusions of midazolam and dexmedetomidine were given over 8h prior to CLP.  Plasma samples were taken a 0h, 2h, 4h, and 5h post insult.  The spleen was resected at the end of the study for analysis.  The study lasted 32h. | 32h mortality  Serum TNF-alpha and IL-6  Splenic caspase-3 | | | Mortality was reduced to a similar degree in both the midazolam and dexmedetomidine groups.  Both sedatives reduced serum levels of TNF-alpha, but only dexmedetomidine reduced levels of IL-6 (although not statistically significant).  Splenic caspase 3 (a marker of apoptosis) was reduced the most in the dexmedetomidine group. | The doses of midazolam and dexmedetomidine were thought to be equipotent.  Depth of sedation was not monitored during the study | At a dose of 0.6mg/kg/hr midazolam and 5 micrograms/kg/hr dexmedetomidine, anti-inflammatory effects can be seen in a CLP rat model. Dexmedetomidine may have pronounced action by reducing IL-6 and apoptosis of splenic cells. |
| **Wu 2013** | 40 rats were randomised into 5 groups of 8.  In addition to a sham group and a CLP only group, 3 other groups underwent CLP and were given varying doses of dexmedetomidine bolus (5 micrograms/kg, 10 micrograms/kg, 20 micrograms/kg) | Dexmedetomidine bolus was given intraperitoneally at 0h, 2h, 4h, and 6h post CLP.  Blood samples were taken for inflammatory markers at 0h, 2h, 4h, and 6h  Left lung sampled at 8h, or immediately after animal death. Bronchoalveolar lavage (BAL) was then performed on the right lung.  The study lasted for a total of 24h. | 24h mortality  Serum TNF-alpha, IL-6  BAL: TNF-alpha, IL-6  Lung tissue: TLR4, MyD88, NFkB  Lung histology | | | A dose dependent improvement in 24h mortality was seen with dexmedetomidine treatment.  Only medium and high dose dexmedetomidine had an anti-inflammatory effect on both serum and BAL cytokine measurements.  Only medium and high dose dexmedetomidine showed histological improvement in lung injury score.  This pattern was also present for NFkB activity. | This study involved large bolus doses of dexmedetomidine given intraperitoneally. | Bolus of 10 micrograms/kg and 20 micrograms/kg dexmedetomidine may have both a systemic and lung anti-inflammatory effect and 24h mortality benefit in a rat model of CLP. |
| **Xu 2013** | 40 mice were randomised into 4 groups of 10.  In an addition to a sham group and a CLP only group, one group underwent CLP 1h after administration of 40 micrograms/kg dexmedetomidine, and a fourth group underwent CLP with same dose dexmedetomidine administration one hour afterwards. | Dexmedetomidine was given as a bolus intraperitoneally.  Intraperitoneal bolus pre and post  Blood samples were taken for cytokine analysis at 6h, 12h, 24h, and 48h.  Lung tissue samples were collected at 24h and 48h after injury.  This study lasted 7 days. | 7 day mortality  Serum TNF-alpha, IL-6, and HMGB1.  Lung tissue mRNA (HMGB1) | | | Both dexmedetomidine groups showed reduced mortality at 7 days in the pre-treatment group. However, the beneficial effect was most marked in the pre-treatment group.  Cytokine levels were most attenuated at 24 hours post CLP with dexmedetomidine treatment. This was only slightly more marked in the pre-treatment group.  Serum HMGB1 and HMGB1 mRNA (lung) both followed the same pattern as the serum cytokine expression. | This study involved a single bolus of 40 micrograms/kg of dexmedetomidine.  Administration pre and post-CLP was tested. | Time dependent effect- increased if DEX given prior to insult  High doses of DEX  Given as bolus not infusion  Cytokine level returns to control level at 48hrs in all groups |
| **Zhang 2019** | Male mice were divided into five groups.  In addition to the control and sham group (who received a laparotomy only), one group had DEX at 0.1mg/kg, one group had DEX at 0.3mg/kg and one group had DEX AT 0.5MG/KG. | DEX was given as an intravenous bolus 30 minutes after CLP.  Unclear length of study | Histopathology of lung tissue  Estimation of mitochondrial MDA content  Serum IL-6, IL-1b, TNF-alpha | | | **Histopathology:** Significant decrease in lung injury score when DEX used compared to the negative controls.  **Inflammatory cytokines:** DEX attenuated serum inflammatory cytokines  **Mitochondrial function:** DEX significantly attenuated OPA1, Mfn1 and Mfn2 expression in lung tissues of mice with CLP induced injuries. | No specific mention of dose dependent effects, through the higher doses appeared to have a greater attenuating effect.  Unclear numbers and duration of study | Dexmedetomidine given as a post CLP bolus improved lung injury score and attenuates serum inflammatory cytokines. |
| **Zhang 2015** | 48 rats were randomised to 6 groups of 8.  In addition to a sham and CLP only group, two groups underwent CLP with varied dose of dexmedetomidine (5 micrograms/kg, and 10 micrograms/kg), another group underwent CLP with 10 micrograms/kg dexmedetomidine combined with yohimbine, and a final group underwent CLP with yohimbine treatment only. | Dexmedetomidine was given as an intravenous bolus after CLP.  The study lasted 6 hours, after which blood samples, bronchoalveolar lavage, and lung tissues were sampled. | TNF-alpha  IL-6  TLR4  MyD88  NFkB | | | Dexmedetomidine attenuated levels of all measured cytokines in both serum and BAL fluid at both doses.  Both doses of dexmedetomidine also inhibited NFkB activation to a similar degree.  This anti-inflammatory effect was sustained even when co-administered with yohimbine. | The results of this study are outwith the context of other similar experiments.  There was no dose-dependent anti-inflammatory effect seen with dexmedetomidine, and no inhibitory effect shown with yohimbine.  The analysis of MyD88 and NFkB may show dexmedetomidine’s action within this inflammatory cascade involving TLR4 which then results in reduced IL-6 and TNF-alpha. | Intravenous bolus of 5 micrograms/kg or 10 micrograms/kg of dexmedetomidine after CLP shows reduction in systemic cytokine levels which is not dose dependent.  This anti-inflammatory action may not be related to alpha-2 receptor action. |
| **Zhang 2017** | 65 rats were randomised into 5 groups of 10-15.  In addition to a control group, sham group, and CLP only group, a group underwent CLP and dexmedetomidine (10 micrograms/kg), and a fifth group underwent CLP, and co-administration of dexmedetomidine (10 micrograms/kg) with the alpha-2 antagonist atipamezole (1mg/kg). | Dexmedetomidine and atipamezole were given as intraperitoneal bolus prior to CLP.  Blood samples were taken at 0h, 4h, 8h, 12h, and 24h.  The study lasted 24h after which the animals were sacrificed and lung tissue samples taken. | 24h mortality  Lung injury score and lung histology  Arterial blood gases  Caveolin-1 expression | | | Dexmedetomidine markedly improved the survival of rats undergoing CLP.  This improvement was also reflected in lung histology and lung injury score.  Atipamezole blunted this anti-inflammatory effect significantly. | This study used a large dose (10 micrograms/kg) of intraperitoneal bolus dexmedetomidine prior to injury.  No inflammatory markers (serum or lung tissue) were measured.  Mortality was only taken up until 24h.  No cardiovascular parameters were measured during the study. | An intraperitoneal bolus of 10 micrograms/kg dexmedetomidine prior to CLP can increase 24h mortality and reduced lung injury score. This effect may be mediated through the alpha-2 receptor. |
| **IRI** |  |  |  | | |  |  |  |
| **Filos 2012** | 64 rats were randomised into two study arms, both consisting of 4 groups of 8.  In addition to a sham, and a femoral artery haemorrhage only group, another group received clonidine subcutaneous bolus (150 micrograms/kg) 5 times in the 2 days prior to the experiments, and another received the same clonidine regimen and underwent femoral artery haemorrhage. | Animals undergoing haemorrhagic shock were volume resuscitated after 60 minutes of MAP sustained at 30-40mmHg.  3 hours after successful resuscitation, samples were taken including liver, lung, ileum, and blood.  The study lasted 3 days in total. | Arm 1 involved measurements of endotoxin in circulating blood.  Arm 2 involved measurement of superoxide radicals in organ tissues (lung, liver, ileum) | | | Clonidine pre-treatment reduced organic hydroperoxides and superoxide radicals found in all of the tissues sampled.  Circulating endotoxin was also reduced in the clonidine pre-treatment group. | No circulating cytokines were measured in this study  Mortality was not measured | Regular subcutaneous pre-treatment with 150 micrograms/kg of clonidine may reduce production of organic hydroperoxides and superoxide radicals along with circulating endotoxin in a rat model of haemorrhagic shock. |
| **Shen 2013** | 36 rats were randomised into 6 groups of 6.  In addition to a sham and control group, 2 further groups underwent ischaemia-reperfusion injury in addition to different doses of dexmedetomidine infusion (2.5 micrograms/kg/hr and 5 micrograms/kg/hr). Another group underwent IRI alongside the higher dexmedetomidine dose co-administered with yohimbine. A final group underwent IRI and was administered yohimbine only. | IRI was performed by clamping of the superior mesenteric artery for 1h and then declamping.  Dexmedetomidine infusions were given intravenously 1hr prior to IRI.  Yohimbine was given immediately prior to dexmedetomidine and before IRI.  Arterial blood gases were taken immediately following reperfusion.  Lung tissue and BAL samples were taken at 2h  The study lasted 4h. | Lung histology and wet/dry ratio  BAL fluid IL-6 and TNF-alpha  Lung tissue TLR4/MyD88 and NFkB | | | **Lung histology:** A dose dependent improvement was seen in the dexmedetomidine groups. In particular, there was less oedema and inflammatory cell infiltration.    **Lung wet/dry ratio:** This was reduced significantly in both the low and high dose dexmedetomidine groups.    **Lung tissue:** There was a dose-dependent reduction in TLR4/MyD88 and NFkB activity in the dexmedetomidine groups.  **BAL fluid:** Dexmedetomidine reduced the concentrations of I-6 and TNF-alpha. This was dose-dependent. | This study used an infusion of dexmedetomidine 1hr prior to IRI.  The doses used were relatively similar to those used clinically.  Measurements were only taken from lung tissue and BAL fluid.  The anti-inflammatory effect of dexmedetomidine was not affected by yohimbine administration. This is outwith the evidence produced by similar studies. | Pre-treatment with dexmedetomidine can reduce lung injury and inflammation in a model of IRI in rats. |
| **Sugita 2013** | 30 rats were randomised into 5 groups of 6.  In addition to a control group, one group were anaesthetised with pentobarbital (10mg/kg/h) and underwent renal ischaemia reperfusion injury. 2 other groups were anaesthetised with dexmedetomidine (10 micrograms/kg/hr and 20 micrograms/kg/hr) and underwent IRI. A final group was anaesthetised with dexmedetomidine (1 microgram/kg/hr) and pentobarbital. | Intravenous infusions were started at the time of reperfusion.  6 hours after reperfusion was started, the animals were sacrificed and blood samples taken via cardiac puncture. Kidneys were then immediately resected for analysis.  The study lasted 7 hours in total. | | Plasma TNF-alpha, IL-6, iNOS  Cardiovascular physiology  Serum creatinine | **Cardiovascular physiology:** Dexmedetomidine showed an improved MAP in animals after reperfusion injury. Bradycardia was noted at the higher dexmedetomidine dose.  **Creatinine:** This was significantly reduced with both doses of dexmedetomidine, along with combined dexmedetomidine and pentobarbital.  **Inflammatory markers:** Dexmedetomidine administration had no effect on inflammatory marker levels at any of those doses or drug combinations. | | This study used two different large doses of dexmedetomidine infusion,  Cardiovascular parameters were measured, however renal blood flow specifically was not.  The lack of anti-inflammatory effect on serum cytokines is outwith the majority of other similar literature.  The very low dose dexmedetomidine (1 microgram/kg/hr) in combination with pentobarbital showed a similar profile of results to even the highest 20 microgram/kg/hr dose.  No alpha-2 antagonist was used to clarify if the potential renal protective effects are alpha-2 mediated. | Infusion of dexmedetomidine with a dose from 1 microgram/kg/hr to 20 micrograms/kg/hr may improve cardiovascular status and renal injury in a rat model of IRI. |
| **Uysal 2012** | 80 rats were randomised into 4 groups of 20. In addition to a control group, and an IRI only group, 2 further groups underwent IRI (via clamping of femoral artery and vein) and treatment with dexmedetomidine at either 10 micrograms/kg, or 30 micrograms/kg.  Half of each group was studied for 12h, after which they were sacrificed. The other half were followed up for 7 days. | IRI was induced via femoral artery and vein clamping for 12h.  Dexmedetomidine was given as an intravenous bolus at 12h when the femoral clamp was removed.  An epigastric island skin flap was created for further tissue analysis  Half of the group was sampled at 7h, and the other half sampled at 7 days.  The study lasted a total of 7 days. | | Histology  Tissue NO  Tissue malondialdehyde (MDA)  Tissue myeloperoxidase (MPO)  Skin survival | Dexmedetomidine reduced measured tissue levels of NO, malondialdehyde, and myeloperoxidase at both 12h and 7 days.  Malondialdehyde specifically showed a dose dependent reduction at 12h.  Epigastric island skin flap necrosis was globally reduced by both doses of dexmedetomidine. | | This study’s main objective was to assess flap survival with use of dexmedetomidine. All samples are therefore taken from an area of epigastric skin, as opposed to any systemic samples.  Large bolus doses of dexmedetomidine were used immediately after reperfusion was initiated. | Bolus dose of either 10 micrograms/kg or 30 micrograms/kg with dexmedetomidine at time of reperfusion may increase survival of a skin flap at 7 days. |
| **Zhang 2017** | 70 adult Sprague Dawley rats were randomised into 5 groups of 14.  In addition to a sham group, and an IRI only group, one group underwent IRI and dexmedetomidine infusion (6 micrograms/kg/hr for the first 10 mins, then 0.7 micrograms/kg/hr for the subsequent 15 mins), another group underwent the same but with co-administration of yohimbine (12mg/kg/hr for 5 mins, then 0.5 micrograms/kg/hr for the subsequent 20 mins), and a final group underwent IRI and yohimbine treatment only. | IRI was induced by occlusion of the left anterior descending coronary artery for 30 mins, prior to reperfusion for 120 mins. After this, the LAD was re-occluded and tissue samples taken.  Dexmedetomidine infusions were administered and completed prior to IRI.  Yohimbine infusions were administered prior to dexmedetomidine and IRI.  Blood samples were taken immediately after reperfusion was started.  The study lasted 3h in total. | | Myocardial histology  Serum and myocardial IL-6, TNF-alpha  Myocardial HMGB1, TLR4, MyD88 | **Myocardial histology and infarct size:** Pre-treatment with dexmedetomidine significantly reduced infarct size in comparison to the IRI only group. Yohimbine co-administration attenuated this reduction in infarct size.  Dexmedetomidine led to more intact myocardial fibres with less disarrangement compared to IRI only. Yohimbine significantly reduced this protective effect.  **Inflammatory markers:**  Dexmedetomidine pre-treatment attenuated measurements of all inflammatory markers. This anti-inflammatory effect was suppressed by yohimbine. HMGB1 was reduced by dexmedetomidine, as well as the downstream signals MyD88 and TLR4. This effect was again attenuated by co-administration of yohimbine. | | 10 animals died prior to experimental endpoint thought possibly due to excessively deep anaesthesia, ventilator induced lung injury, or cardiac compromise due to the IRI.  Tissue samples only specific to the myocardium were taken and assessed.  The loading and infusion regimen for both study drugs could be thought to be more clinically applicable compared to large bolus doses.  Dexmedetomidine was used as a pre-conditioning drug for the myocardium, given prior to IRI. | Pre-treatment with dexmedetomidine infusion may reduce infarct size and histological evidence of myocardial injury in a rat model of IRI. This effect may be mediated via the alpha-2 receptor. |
