# Supplementary material for: The effect of dexmedetomidine and clonidine on the inflammatory response in critical illness: a systematic review of animal and human studies
Source: Crit Care. 2019 Dec 11;23:402. doi: 10.1186/s13054-019-2690-4 (PMC6907244; doi:10.1186/s13054-019-2690-4)
Supplement: Supplementary file 4 — Additional file 4. Summary of human studies. [file 13054_2019_2690_MOESM4_ESM.doc]

| **Author** | **Population** | **Intervention** | **Key Findings** | **Conclusions** |
| --- | --- | --- | --- | --- |
| Gao 2015 | RCT of 50 patients undergoing elective lobectomy and one lung ventilation | Dexmedetomidine bolus 1 microgram/kg prior to general anaesthesia | **1.**Dexmedetomidine was not associated with any changes in HR, MAP, or Bispectral Index (BIS).  **2.** Dexmedetomidine was associated with reduced serum TNF-α and MDA at 60 minutes and 90 minutesInflammatory markers: The group given dexmedetomidine showed reduced TNF-α and MDA at 60 min and 90 min after one lung ventilation. | Pre-treatment with 1 microgram/kg of dexmedetomidine prior to anaesthesia was associated with reduced biochemical markers of lung injury after lobectomy and one lung ventilation. |
| Kang 2013 | RCT of 47 patients undergoing elective laparoscopic cholecystemomy for chronic cholecystitis. | Dexmedetomidine 1 microgram/kg laoding dose and 0.5 microgram/kg infusion was started immediately after induction of general anaesthesia. | **1.**Dexmedetomidine was associated with lower requirement for sevoflurane and remifental intra-operatively.  **2.** Dexmedetomidine was associated with reduced intra-operative fentanyl requirement.  **3.** Dexmedetomidine was associated with reduced intra-operative cytokines (below baseline). Notably, the control group had no increase in cytokines to suggest an inflammatory response to surgery.  **4.** Dexmedetomidine was associated with reduced CRP and white cell count (WCC) day 1 post-operatively. | Intra-operative dexmedetomidine infusion at 0.5 micrograms/kg/hr was associated with reduced intra-operative cytokines and reduced WCC and CRP day 1 post-operatively in patients receiving elective laparoscopic cholecystectomy. |
| Kawazoe 2017 | Multi centre RCT of 201 ventilated ICU patients with a diagnosis of sepsis. | **Control:** Propofol, midazolam, and analgesia  **Treatment:** Dexmedetomidine and analgesia+/- other sedation if required  Sedative agents were titrated to target sedation score in the ICU. | **Primary outcomes:** This study demonstrated an 8% reduction in 28 day mortality with dexmedetomidine sedation – however, this did not reach statistical significance. There was no difference in number of ventilator free days.  **Secondary Outcomes:** Only CRP showed a statistically significant reduction in the dexmedetomidine group. | Use of dexmedetomidine as a primary sedative agent for ventilated patients diagnosed with sepsis shows no significant difference in 28 day mortality of number of ventilator free days. |
| Memis 2007 | RCT of 40 ventilated ICU patients with a diagnosis of bacterial sepsis. | **Control:** Midazolam (0.2mg/kg loading +  0.1-0.5mg/kg/hr maintenance) + alfentanil.  **Treatment:** Dexmedetomidine (1micrograms/kg loading + 0.2-2.5micrograms/kg/hr maintenance) + alfentanil.  Infusion rate was titrated within the maintenance range to achieve a RASS score >2. | **1**.There was no difference in haemodynamic measurements between the two groups.  **2**.Dexmedetomidine was associated with a statistically significant reduction in all measured cytokines at 24h.  **3.**No difference in gastric pH was found between the two groups. | Dexmedetomidine infusion 0.2-2.5 micrograms/kg/hr was associated with reduced serum cytokines after 24h. |
| Tasdogan 2009 | RCT of 40 ventilated ICU patients.  All patients were post-ileal surgery and had scored at least 2 sepsis criteria. | **Control:** Propofol sedation (loading dose of 1mg/kg, followed by a maintenance infusion of 3mg/kg/hr).  **Treatment:** The dexmedetomidine group received a loading dose of 1 microgram/kg, followed by a maintenance infusion of 0.2-2.5 micrograms/kg/hr. | **1.** There was no significant difference in BP, HR, MAP, and urine output between the two study groups.  **2.** TNF-α, IL-1, and IL-6 were all significantly reduced in the dexmedetomidine group at 24h, and 48h. | A 24h dexmedetomidine infusion of 0.2-2.5 micrograms/kg/hr in mechanically ventilated patients treated for sepsis after ileus surgery was associated with reduced systemic cytokine levels up to 48h. |
| Ueki 2014 | RCT of 37 patients undergoing elective cardiac surgery involving cardiopulmonary bypass. | Dexmedetomidine was given as 1 micrograms/kg loading dose and 0.5 micrograms/kg/hr maintenance infusion for the duration of the operation. | **1.**Dexmedetomidine was associated with significantly attenuated HMGB1 levels between 1-4h post-operatively.  **2.** Dexmedetomidine was associated with much quicker return to baseline in serum IL-6.  **3**.NFkB activity was reduced at 4h post-operatively in the dexmedetomidine group.  **4**.There was a statistically significant post-operative reduction in serum AST and CRP in the dexmedetomidine group. | An intra-operative infusion of 0.5 micrograms/kg/hr of dexmedetomidine may reduce post-operative cytokine levels in patients undergoing elective cardiac surgery involving cardiopulmonary bypass. |
| Venn 2001 | RCT of 20 ventilated ICU patients post-elective abdominopelvic surgery. | **Control:** propofol sedation given as 1mg/kg loading dose plus 3mg/kg/hr maintenance infusion.  **Treatment:** Dexmedetomidine sedation given as 2.5 micrograms/kg/hr loading dose plus 0.2-2.5 micrograms/kg/hr maintenance infusion.  Sedation was titrated to a target RASS score >2. | **1.** Patients receiving dexmedetomidine had significantly lower heart rates in comparison to the propofol group. There was no difference in blood pressure or MAP between the groups.  **2.** There was no difference found in cortisol or ACTH concentrations between the groups.  **3.**There was no difference in any other inflammatory or endocrine factors measured with the exception of a significant increase in growth hormone in the dexmedetomidine group. | Dexmedetomidine infusion for sedation at 0.2 – 2.5 micrograms/kg/hr was associated with more bradycardia compared to propofol. It showed no difference in circulating inflammatory or endocrine markers, with the exception of raised growth hormone. |
| Yongsuk 2014 | RCT of 46 patients undergoing elective laparoscopic cholecystectomy. | Dexmedetomidine was given as a 1 microgram/kg loading dose, followed by 0.5 micrograms/kg/hr maintenance infusion for the duration of the operation. This was started immediately following induction of general anaesthesia. | **1.**Intra-operative IFN gamma was lower during the operation in the saline group in comparison to a normal steady state in the dexmedetomidine group.  **2.** The IFN gamma/IL-4 ratio (Th1 cytokine: Th2 cytokine) was higher in the dexmedetomidine group. | Intra-operative dexmedetomidine infusion of 0.5 micrograms/kg/hr may increase IFN gamma;IL 4 ratio within the peri-operative period. This may suggest dexmedetomidine’s immunomodulatory effect is to shift the Th1:Th2 ratio towards Th1. |
| Zhou 2017 | RCT of 40 patients undergoing elective multilevel spinal fusion operations. | Dexmedetomidine was given as 0.5 micrograms/kg loading dose plus 0.5 micrograms/kg/hr maintenance infusion throughout the operation.  This was started prior to induction of general anaesthesia. | **1.**The dexmedetomidine group showed significant reduction in post-operative white cell count (WCC) and CRP.  **2.**TNF-α and IL-6 levels were also lower in the dexmedetomidine group up until post-operative day 3.  **3.**CD42a/CD14+ was globally reduced in the dexmedetomidine group.  **4.**HLADR+/D14 was globally increased in the dexmedetomidine group. | An intra-operative infusion of 0.5 micrograms/kg/hr of dexmedetomidine may reduce post-operative systemic cytokines in patients undergoing elective multilevel spinal fusion surgery. |
