# Supplementary material for: The effect of dexmedetomidine and clonidine on the inflammatory response in critical illness: a systematic review of animal and human studies
Source: Crit Care. 2019 Dec 11;23:402. doi: 10.1186/s13054-019-2690-4 (PMC6907244; doi:10.1186/s13054-019-2690-4)
Supplement: Supplementary file 5 — Additional file 5. Detailed description of human studies. [file 13054_2019_2690_MOESM5_ESM.doc]

| **Author** | **Population** | **Intervention** | | | **Outcome**  **measures** | **Key Findings** | **Bias** | **Comments** |
| --- | --- | --- | --- | --- | --- | --- | --- | --- |
| Gao 2015 | 50 patients were randomised to a control group and dexmedetomidine treatment group (n=25 each).  These patients all underwent elective lobectomy procedures and were given one lung ventilation intra-operatively. | Patients received 1 microgram/kg of dexmedetomidine prior to induction of general anaesthesia (maintained by propofol and remifentanil total intravenous anaesthesia)  Blood samples were taken at 0h (before anaesthesia), and at 30 min, 60 min, and 90 min after one lung ventilation.  Abnormal lung tissue was resected 5cm away from the tumour. | | | BP, MAP, HR and Bispectral Index (BIS)  Serum TNF-alpha, malondialdehyde (MDA), and superoxide dismutase (SOD).    Lung tissue heme-oxygenase-1 (HO-1) | **Physiological parameters:** The group receiving dexmedetomidine showed no changes in blood pressure, MAP, heart rate, and BIS compared to the control group. Duration of one lung ventilation and time to resection were also similar.  **Inflammatory markers:** The group given dexmedetomidine showed reduced TNF-alpha and MDA at 60 min and 90 min compared to the control group.  SOD and HO-1 levels were much higher in the dexmedetomidine group at each time point. | This study involved a relatively small number of patients  This work was done in a single centre  Patient outcome factors were such as mortality and surgical complication rate were not measured. | Pre-treatment with 1 microgram/kg of dexmedetomidine prior to anaesthesia may reduce biochemical markers of lung injury after lobectomy and one lung ventilation. It may do this by increasing levels of HO-1. |
| Kang 2013 | 47 patients undergoing elective laparoscopic cholecystectomy for chronic cholecystitis were randomised into a control group or a dexmedetomidine group (n=23, n=24 respectively) | Dexmedetomidine was administered immediately after induction of general anaesthesia.  A loading dose of 1 microgram/kg was given over 10 minutes, following by an infusion of 0.5 micrograms/kg/hr for the duration of the procedure.  Blood samples were taken at peritoneal closure and at 60 min after the end of surgery. | | | BP, HR, MAP, anaesthetic dose required, intra-operative analgesia required.  White cell count (WCC), CRP  Plasma IL-1b, IL-6, IL-10, IL-4, and TNF-alpha | **Clinical observations:** The patients receiving dexmedetomidine infusion had lower requirement of sevoflurane and remifentanil to maintain anaesthesia. They also had lower intra-operative fentanyl requirement for analgesia. Mean duration of surgery and anaesthesia was comparable between the two groups.  **Plasma cytokines:** Cytokine levels did not increase in the control group during surgery. In the dexmedetomidine group, they declined below baseline. This was statistically significant for IL-1b, TNF-alpha, and IL-10.  **CRP/WCC**: These were both lower in the dexmedetomidine group on day 1 post-operatively. | The cytokine pattern was not increased during surgery, which is inconsistent with previous literature. This may be due to the more minor stimulus from laparoscopic surgery, or the use of known anti-inflammatory agents such as remifentanil and sevoflurane.  The anaesthetic-sparing effect of dexmedetomidine may have played a part in the reduced cytokine profile. | Administration of dexmedetomidine after induction of general anaesthesia may reduce intra-operative levels of systemic cytokines. |
| Kawazoe 2017 | Open label, multi centre randomised controlled trial.  201 ventilated ICU patients with sepsis were randomised to either 1) standard care sedation (Propofol, midazolam, and analgesia) or 2) treatment group (dexmedetomidine and analgesia +/- other sedative agents as needed) | | Sedative agents were titrated to target sedation score in the ICU.  No antagonist used  Daily blood samples  Study length= mechanical ventilation/as needed | **Primary Outcomes:** 28 day mortality and number of ventilator free days  **Secondary Outcomes:**  CRP  Procalcitonin  Prealbumin  Disseminated Intravascular Coagulation score | | **Primary outcomes:** This study demonstrated an 8% reduction in 28 day mortality with dexmedetomidine sedation – however, this did not reach statistical significance. There was no difference in number of ventilator free days.  **Secondary Outcomes:** Only CRP showed a statistically significant reduction in the dexmedetomidine group. | The administered dose of dexmedetomidine was lower in this trial than many other countries use due to the lower dosage limit covered by Japanese medical insurance.  The subjects in this study had a mean age of 69, with a mean APACHE score of 23. This means they were older, and less sick, than groups from similar studies.  This was an open label study with endpoints assessed by physicians at discharge.  The study was underpowered to detect the 8% mortality difference seen.  No long term outcomes were assessed. | Use of dexmedetomidine as a primary sedative agent for ventilated patients diagnosed with sepsis shows no significant difference in mortality of number of ventilator free days. |
| Memis 2007 | 40 patients mechanically ventilated patients with a diagnosis of bacterial sepsis were randomised to receive either midazolam and alfentanil for sedation, or dexmedetomidine and alfentanil as sedation. | | Midazolam was administered as 0.2mg/kg loading +  0.1-0.5mg/kg/hr maintenance.  Dexmedetomidine was administered as 1micrograms/kg loading + 0.2-2.5micrograms/kg/hr maintenance.  Infusion rate was titrated within the maintenance range to achieve a RASS score >2.  A nasogastric tonometer was inserted to determine gastric pH  Blood samples were taken at baseline and 24h | Cardiovascular physiology including BP, MAP, and HR  Gastric pH  Plasma TNF-alpha, IL-1b, and IL-6. | | There was no significant difference in haemodynamic measurements between the two groups during the study period.  Patients randomised to dexmedetomidine sedation showed a statistically significant reduction in all measured cytokines at 24h. No reduction was seen in the midazolam group.  No difference in gastric pH was found between the two groups. | This study involved small patient numbers.  No measurement of clinical outcome was involved eg mortality, daily SOFA score.  The study period only lasted 24h.  5 patients died within 24h of randomisation due to septic shock | Dexmedetomidine infusion 0.2-2.5 micrograms/kg/hr may be associated with reduced systemic cytokines after 24h. |
| Tasdogan 2009 | Prospective, single-centre study of 40 ICU patients.  Patients recruited were post-operative from ileus surgery who had scored at least 2 sepsis criteria.  One group were randomised to primary propofol sedation or primary dexmedetomidine sedation. | | The propofol group received a loading dose of 1mg/kg, followed by a maintenance infusion of 3mg/kg/hr.  The dexmedetomidine group received a loading dose of 1 microgram/kg, followed by a maintenance infusion of 0.2-2.5 micrograms/kg/hr.  Infusions were given over 24h  Blood samples were taken at 0h, 24h, and 48h. | Cardiovascular physiology  Plasma TNF-alpha, IL-1b, and IL-6 | | **Cardiovascular physiology:** There was no significant difference in BP, HR, MAP, and urine output between the two study groups.  **Inflammatory markers:** TNF-alpha, IL-1, and IL-6 were all significantly reduced in the dexmedetomidine group at 24h, and 48h. | This study involved a small number of patients recruited in a single centre.  No longer term clinical outcome was measured. | A 24h dexmedetomidine infusion of 0.2-2.5 micrograms/kg/hr in mechanically ventilated patients treated for sepsis after ileus surgery may reduce systemic cytokine production up to 48h. |
| Ueki 2014 | Single centre, prospective, randomised controlled trial.  37 patients undergoing elective cardiac surgery involving cardiopulmonary bypass were randomised to either place or dexmedetomidine treatment intra-operatively. | | Patients receiving dexmedetomidine were given 1 micrograms/kg loading dose and 0.5 micrograms/kg/hr maintenance infusion for the duration of the operation.  Blood samples taken from anaesthetic induction, after aortic occlusion, 1h after declamping aorta, 4h after declamping aorta, and post-operative day 1 and 3.  Study length= 3 days | Plasma CRP, IL-6, HMGB1, and NFkB | | Dexmedetomidine significantly attenuated HMGB1 levels between 1-4h post-op, after which levels returned back to baseline.  IL-6 levels returned to baseline much faster in the dexmedetomidine group.  NFkB activity was reduced at 4h post-operatively in the dexmedetomidine group.  There was a statistically significant post-operative reduction in serum AST and CRP in the dexmedetomidine group. | This was a single centre study that involved 37 elective cardiac patients.  No longer term clinical outcomes were measured for each patient.  All patients were routinely given steroid therapy peri-operatively. | An intra-operative infusion of 0.5 micrograms/kg/hr of dexmedetomidine may reduce post-operative cytokine levels in patients undergoing elective cardiac surgery involving cardiopulmonary bypass. |
| Venn 2001 | Single centre, randomised controlled trial.  20 patients admitted to ICU after elective abdomino-pelvic surgery were randomised to receive either standard care sedation (propofol +/- alfentanil) or dexmedetomidine +/- alfentanil sedation. | | The propofol group were given 1mg/kg loading dose plus 3mg/kg/hr maintenance infusion.  The dexmedetomidine group were given 2.5 micrograms/kg/hr loading dose plus 0.2-2.5 micrograms/kg/hr maintenance infusion.  Sedation was titrated to a target RASS score >2.  Blood samples were taken at 2h,4h,6h, 8h,12h, and 24h post-operatively. | Cardiovascular physiology  Endocrine function: serum cortisol, ACTH, short synacthen test  Plasma Il-6, glucose, growth hormone, insulin, prolactin | | **Cardiovascular physiology:** Patients receiving dexmedetomidine had significantly lower heart rates in comparison to the propofol group. There was no difference in blood pressure or MAP between the groups.  **Endocrine function:** There was no difference found in cortisol or ACTH concentrations between the groups.  There was no difference in any other inflammatory or endocrine factors measured with the exception of a significant increase in growth hormone in the dexmedetomidine group. | This was a small, single centre study of 20 ICU patients with little statistical power to detect much difference in measured inflammatory or endocrine markers. | Dexmedetomidine infusion for sedation at 0.2 – 2.5 micrograms/kg/hr was associated with more bradycardia compared to propofol. It showed no difference in circulating inflammatory or endocrine markers, with the exception of raised growth hormone. |
| Yongsuk 2014 | Single centre, randomised, placebo controlled trial  46 patients undergoing elective laparoscopic cholecystectomy were randomised to receive either placebo or dexmedetomidine | | Immediately following anaesthetic induction, the dexmedetomidine group were given a 1 microgram/kg loading dose, followed by 0.5 micrograms/kg/hr maintenance infusion for the duration of the operation.  Blood samples at induction, peritoneal closure and 1h post-operatively. | Interferon (IFN) gamma  IL-4 | | IFN gamma lowered during the operation in the saline group in comparison to a normal steady state in the dexmedetomidine group.  The IFN gamma/IL-4 ratio (Th1 cytokine: Th2 cytokine) was higher in the dexmedetomidine group. | This was a small, single centre study of elective laparoscopic patients.  Baseline cytokine assay samples were taken after induction of anaesthesia. Many anaesthetic drugs may play a role in altering this profile.  IFN gamma:IL-4 ratio is used as a surrogate to determine Th1:Th2 shift  No longer term clinical outcomes were measured. | Intra-operative dexmedetomidine infusion of 0.5 micrograms/kg/hr may increase IFN gamma; IL 4 ratio within the peri-operative period. This may suggest dexmedetomidine’s immunomodulatory effect is to shift the Th1:Th2 ratio towards Th1. |
| Zhou 2017 | Single centre, randomised, placebo controlled trial  40 patients undergoing elective multilevel spinal fusion operations were randomised to receive either placebo or dexmedetomidine infusion intra-operatively. | | The dexmedetomidine group received 0.5 micrograms/kg loading dose plus 0.5 micrograms/kg/hr maintenance infusion throughout the operation.  Loading dose and infusion were given prior to anaesthetic induction.  Blood samples were taken at baseline, 30min, 60min, 1 day, 3 days, 5 days post operatively. | CRP + white cell count (WCC)  IL-6 + TNF-alpha  IL-10  CD42a/CD14+  HLADR/CD14+ | | The dexmedetomidine group showed significant reduction in post-operative white cell count (WCC) and CRP.  TNF-alpha and IL-6 levels were also lower in the dexmedetomidine group up until day 3 post-operatively.  CD42a/CD14+ was globally reduced in the dexmedetomidine group.  HLADR+/D14 was globally increased in the dexmedetomidine group. | This was a small, single centre clinical trial in patients undergoing elective surgery.  No longer term clinical outcomes were used | An intra-operative infusion of 0.5 micrograms/kg/hr of dexmedetomidine may reduce post-operative systemic cytokines in patients undergoing elective multilevel spinal fusion surgery. |
